# Nucleic sexual hormone receptor signaling pathways in breast cancer: function regulation, crosstalk, and therapeutic implications

**DOI:** 10.1038/s41698-026-01568-x

**Published:** 2026-06-22

**Authors:** Kai Zeng, Anqi Wang, Hongmiao Sun, Chunyu Wang, Shengli Wang, Renlong Zou, Dongjun Yang, Yu Bai, Manlin Wang, Yingying Xu, Yue Zhao

**Affiliations:** 1 Department of Cell Biology, Key Laboratory of Cell Biology, Ministry of Public Health, and Key Laboratory of Medical Cell Biology, Ministry of Education, School of Life Sciences, China Medical University, Shenyang, Liaoning Province China; 2 Department of Breast Surgery, The First Hospital of China Medical University, Shenyang, Liaoning Province China

**Keywords:** Cancer, Cell biology, Endocrinology, Molecular biology

## Abstract

Sexual hormone receptors (SHRs) are essential for breast cancer (BCa) pathogenesis. BCa is a prevalent malignancy with high heterogeneity and high recurrence. Endocrine resistance remains a major clinical challenge. SHR-mediated transcription involves complex epigenetic, post-translational, and inter-receptor crosstalk, and dysregulation of these processes contributes to endocrine resistance. This review aims to summarize the current progress on the molecular mechanism underlying the function of nucleic SHRs in BCa, providing insights for the novel therapeutic strategies in BCa.

## Introduction

Breast cancer is a biologically heterogeneous disease with complex molecular foundations, and its clinical management continues to evolve in response to persistent challenges such as recurrence and therapeutic resistance^1^. It remains the most commonly diagnosed malignancy among women, accounting for an estimated 32% of new cancer cases in 2025. Despite a 44% decline in mortality since 1989, BCa is still the second leading cause of cancer-related death in women, with ~42,680 projected deaths^2^.

Sexual hormone receptors, including estrogen receptor (ER), progesterone receptor (PR), and androgen receptor (AR), govern transcriptional programs central to BCa cell proliferation, differentiation, and survival^3^. These receptors serve not only as biomarkers for disease classification but also as therapeutic targets, particularly in hormone receptor-positive subtypes^4–6^. As members of the steroid nuclear receptor family, they share conserved structural features and mechanisms of action. Each receptor is activated by its cognate ligand: estrogen binds ER, progesterone binds PR, and androgen binds AR^7^.

Clinically, tumors expressing ER and/or PR are classified as hormone receptor-positive BCa, whereas those lacking ER, PR, and human epidermal growth factor receptor 2 (HER2) expression are defined as triple-negative breast cancer (TNBC)^8^. Approximately 70% of BCa are ER-positive, and their development is closely linked to aberrant activation of ER signaling. As a result, numerous endocrine therapies have been developed to target the estrogen-ER axis, forming the cornerstone of treatment for ER-positive disease^9^.

Recent studies, however, have highlighted the broader significance of PR and AR signaling, as well as their crosstalk with ER pathways, in shaping BCa biology and influencing therapeutic responses^10,11^. Moreover, transcriptional regulation mediated by sexual hormone receptors is subject to multiple layers of control, ranging from chromatin remodeling to post-translational modifications^12–15^. Given this complexity, simply targeting receptor signaling with endocrine therapy remains insufficient, as resistance frequently emerges^12,16,17^.

In this review, we examine the structural and functional properties of sexual hormone receptors and analyze the multilayered regulation of sexual hormone receptor-mediated transcription in breast cancer. We specifically focus on nuclear genomic pathways, excluding membrane receptor-mediated nongenomic rapid signaling. Particular attention is given to the molecular mechanisms by which epigenetic regulators and post-translational modifications govern ER, PR, and AR activity during transcriptional activation, as well as the inter-receptor crosstalk. We further discuss the therapeutic potential of inhibitors targeting these co-regulators to enhance endocrine efficacy and overcome resistance.

## The structural and functional features of sexual hormone receptors

**Fig. 1:**
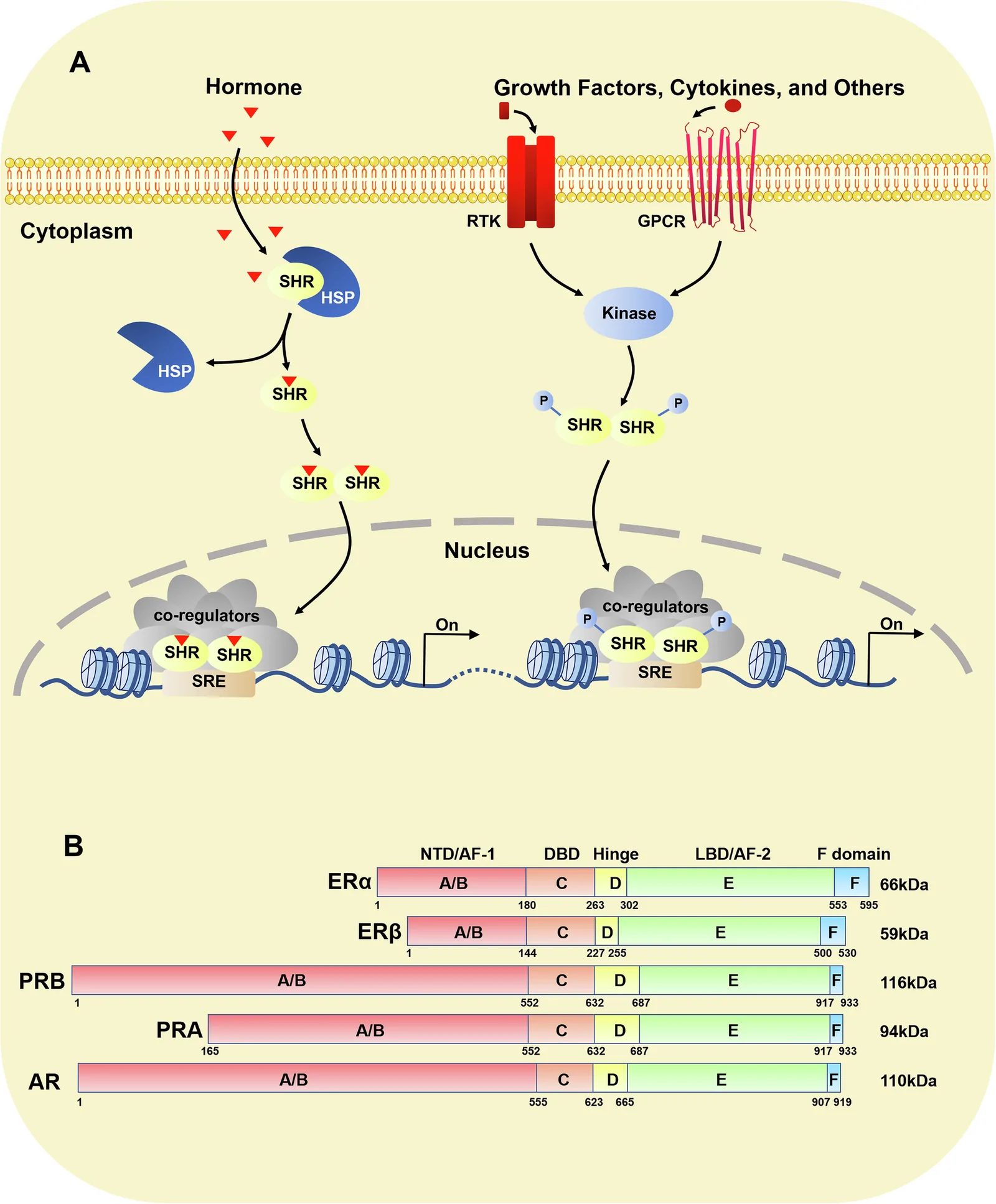
Principal actions and structure of sex hormone receptors. **A** Sex hormone receptors (SHRs), including ER, PR, and AR, are sequestered in the cytoplasm by chaperone complexes (e.g., Hsp90 and Hsp70) in the inactive state. Ligand (Hormone) binding induces conformational changes, chaperone dissociation, receptor dimerization, and nuclear translocation. In the nucleus, activated receptors bind specific DNA response elements (SREs, i.e., EREs for ER, PREs for PR, and AREs for AR) at promoters or distal enhancers, and recruit multiple co-regulators to regulate target gene transcription. Alternatively, extracellular cues, including growth factors and cytokines, engage membrane-associated receptor tyrosine kinases (RTKs) or G-protein-coupled receptors (GPCRs), triggering downstream kinase cascades that phosphorylate and activate cytoplasmic sexual hormone receptors. These activated receptors subsequently translocate into the nucleus in a ligand-independent manner to regulate target gene expression. **B** Schematic illustration of the conserved modular domain architecture shared by ER, PR, and AR. The N-terminal A/B domain (NTD/AF-1) mediates ligand-independent transcriptional activation. The centrally located C domain (DBD) directs sequence-specific DNA binding. The D domain (hinge region) serves as a flexible allosteric switch that harbors the NLS and functions as a regulatory platform for co-regulator recruitment and post-translational modifications. The E domain (LBD/AF-2) serves as the principal hormone-binding interface and drives ligand-dependent activation. The F domain at the extreme C-terminus modulates LBD activity and fine-tunes receptor function.

As classical ligand-activated nuclear receptors, sexual hormone receptors share a conserved modular architecture and common mechanisms governing ligand-dependent activation and target gene transcription. In the inactive state, most receptors remain in the cytoplasm, where they are stabilized by molecular chaperone complexes that include heat shock proteins such as Hsp90 and Hsp70. Upon ligand engagement induces conformational rearrangements and phosphorylation events that promote dissociation from chaperones, receptor homo‑ or hetero‑dimerization, and subsequent nuclear translocation^18,19^. Within the nucleus, activated receptors recognize and bind specific DNA motifs via their DNA binding domain. Collectively termed sexual hormone response elements (SREs), these include estrogen response elements (EREs) for ER, progesterone response elements (PREs) for PR, and androgen response elements (AREs) for AR. These binding sites are widely distributed across the genome; in addition to canonical promoter regions, recent studies have identified extensive occupancy within distal enhancer elements, underscoring the complexity of receptor‑mediated transcriptional regulation^11,20^. Accumulating evidence demonstrates that the transcriptional programs driven by sexual hormone receptors are critically dependent on the recruitment of diverse co‑regulatory factors. These co‑regulators orchestrate chromatin remodeling, modulate receptor transcriptional activity, affect receptor protein stability or receptor-DNA binding, thereby shaping hormone receptor-mediated transcriptional programs in BCa^3,15,19,21^ (Fig. 1A).

ER exist as two subtypes, ERα and ERβ, encoded by the *ESR1* and *ESR2*, respectively. *ESR1* is located on chromosome 6q25.1, whereas *ESR2* is situated on a different chromosome at 14q23.2. ERα comprises 595 amino acids with a molecular weight of 66 kDa, whereas ERβ consists of 530 amino acids and has a molecular weight of 59 kDa. 17β‑estradiol (E2) is the predominant circulating estrogen^22^. Pioneer transcription factors such as FOXA1, GATA3, PBX1, and AP2γ facilitate ERα chromatin occupancy by engaging condensed chromatin^23–26^. However, several clinical studies have shown that *ESR2* promoter methylation is increased in breast cancer compared with normal breast tissue, and that hypermethylation is associated with reduced or even absent ERβ expression in a substantial subset of tumors^27,28^. As a result, the capacity of ERβ to counterbalance ERα is markedly diminished, making aberrant ERα activation the predominant driver of breast cancer progression^29^. PR is encoded by the *PGR* located at 11q22.1 and is frequently upregulated in ERα-positive BCa, consistent with PGR being a direct transcriptional target of ERα^16,30^. Two main isoforms, PRA (94 kDa, 769 amino acids) and PRB (116 kDa, 933 amino acids), are generated from alternative transcription start sites of the single PGR gene. Both isoforms share identical DNA- and ligand-binding domains, but only PRB contains an additional 165-amino-acid N-terminal extension that encodes a potent activation function-3 (AF-3). Progesterone is the endogenous ligand of PR^31^. Androgen receptor (AR) is encoded by *AR* on chromosome Xq12. The full-length protein comprises 919 amino acids with a molecular weight of ~110 kDa. 5α-dihydrotestosterone (DHT) exhibits higher potency than testosterone as the most potent endogenous ligand for AR^32–34^.

Sexual hormone receptors share a common evolutionary origin and display a highly conserved modular architecture (Fig. 1B). The N-terminal domain (NTD; regions A/B) harbors the transcriptional activation function-1 (AF-1), which mediates ligand-independent protein interactions and contributes to receptor-specific transcriptional activation of target genes. The DNA-binding domain (DBD; region C) is highly conserved and essential for DNA recognition. The hinge region (region D), connecting the DBD and ligand-binding domain, contains a nuclear localization signal. Because this region can directly participate in DNA binding, it has also been termed the carboxyl-terminal extension (CTE) of the DBD. The intrinsic flexibility of the hinge/CTE maintains dynamic equilibrium between conformations that alternately repress the core DBD, bind DNA minor grooves flanking response elements, and recruit co-regulatory proteins, functioning as an allosteric switch regulating DNA specificity and transcription^35^. Furthermore, recent studies have demonstrated that the hinge region contains multiple residues subject to post-translational modifications, including phosphorylation, acetylation and methylation. These modifications modulate ligand sensitivity, DNA-binding affinity, receptor stability, and even cytoplasmic localization. Collectively, these findings establish that the hinge/CTE functions not merely as a flexible, unstructured linker between the DBD and LBD, but as an indispensable multifunctional platform.

The ligand-binding domain (LBD; region E) harbors the activation function-2 (AF-2), which mediates ligand-dependent transcriptional activation through recruitment of co-regulatory proteins bearing LXXLL motifs. Upon hormone binding, the dynamic C-terminal helix 12 repositions to form a hydrophobic cleft accommodating these co-activators, whereas antagonist binding disrupts this interaction surface, thereby determining the receptor’s transcriptional output. The F domain, a short and poorly conserved C‑terminal segment located immediately downstream of helix 12, remains incompletely characterized. It has been proposed to function as a conformation-coupled regulatory module that fine‑tunes receptor activity by influencing H12-dependent ligand-binding states, modulating co-regulator interactions, and shaping dimerization behavior in a receptor- and ligand-specific manner^3,18,36–39^. In addition, interspecies sequence variation appears to contribute to distinct ligand responses^40^. Notably, the ERα F domain (553–595) shapes cofactor selectivity by modulating the ligand‑dependent positioning of helix 12, thereby controlling AF‑2 accessibility. It also stabilizes the protein-protein interface required for ERα-Sp1 interactions, thereby enabling target gene transcription at Sp1-binding sites. In addition, the F domain provides a docking interface for 14‑3‑3 proteins, whose association sterically interferes with receptor dimerization and limits coactivator engagement, thereby adding an additional layer of transcriptional repression.^37,41,42^. In relation to SRC-1, truncation mutants of the ERα F domain have yielded distinct outcomes. Koide et al. reported that removal of the last 16 residues reduced responsiveness to SRC‑1, whereas Yang et al. found that deletion of the final 24C‑terminal amino acids enhanced recruitment, indicating that this interaction maybe region‑specific and dependent on cellular context^43,44^. However, the ERβ F domain lacks predicted or observed secondary structure, and its functional properties differ significantly from those of the ERα F domain; for instance, deletion or replacement of the ERβ F domain does not alter ligand activation patterns, whereas the ERα F domain critically regulates transactivation, cofactor interactions, and antagonist activity^37^. The extreme C terminus of PRB, commonly referred to as the F domain (approximately residues 917–933), harbors a transcriptional repressor function. Genetic studies have mapped the minimal repressor element to amino acids 917–928^45^, whereas crystallographic analysis of the liganded PR LBD reveals a structured C-terminal extension spanning residues 922–933 that anchors helix 12 against the LBD body and modulates ligand-dependent protease resistance and dimerization^46^. The overlapping region (922–928) constitutes the functional core that couples antagonist-induced conformational exposure to corepressor recruitment. Although the functional role of the AR F‑domain is not as clearly defined as that of the ERα or PR, this short C-terminal extension immediately following helix 12 (907–919) is nevertheless essential for androgen binding because its hydrophobic and aromatic residues, particularly Phe916, stabilize the proper ligand-binding conformation and thereby maintain receptor activity^37,47^.

Sexual hormone receptors share a conserved modular architecture and a common mode of ligand dependent transcriptional activation, yet their functional outputs in breast cancer are shaped by highly distinct, multilayered regulatory programs. Beyond their structural homology, the transcriptional activities of ERα, PR, and AR are critically dependent on genomic alterations, epigenetic co-regulation, and post translational modifications, all of which are orchestrated by diverse co regulatory factors. These co regulators modulate chromatin remodeling, receptor protein stability, DNA binding affinity, and cofactor recruitment, thereby generating context specific signaling patterns^3,15,19,21^. Furthermore, modest but functionally relevant differences across individual receptor domains introduce selectivity in co-regulator engagement and chromatin recruitment, providing a mechanistic basis for the extensive yet differential crosstalk observed among the three receptors. Consequently, the transition from hormone dependent to hormone independent growth and the emergence of endocrine resistance involve regulatory complexities that extend beyond receptor architecture, underscoring the importance of integrated co regulator networks. In the following sections, we sequentially examine the signaling pathways of ERα, PR, and AR in breast cancer. We further discuss how these co regulatory mechanisms shape endocrine therapeutic efficacy and explore strategies for pharmacologic targeting to overcome resistance.

## Estrogen receptor signaling pathways in BCa

Extensive studies have shown that ERα regulates a broad spectrum of genes implicated in BCa progression, including *TFF1*, *FOXM1*, *MTA3*, *HIF1A*, *GREB1*, *CCND1*, *E2F1*, and *MYC*. In addition, ERα transactivates several non‑coding RNAs, such as miR‑18a and miR‑106a, which further shape its transcriptional program^48–50^. These targets are central to cell‑cycle regulation, stem cell dynamics, and malignant behaviors including tumor growth, invasion, and metastasis^51–56^. Beyond its canonical role as a transcription factor, ERα also functions as a non‑canonical RNA‑binding protein. It promotes XBP1 mRNA splicing, a key step in the unfolded protein response (UPR), and regulates the translation of stress‑response mRNAs such as eIF4G2 and MCL1. Through these post‑transcriptional mechanisms, ERα enhances cancer cell survival under stress and contributes to tamoxifen resistance^57^.

Accordingly, therapeutic strategies targeting ERα signaling form the cornerstone of endocrine therapy. These include selective estrogen receptor modulators (SERMs) such as tamoxifen, which antagonize ERα activity; selective estrogen receptor degraders (SERDs) such as fulvestrant, which promote ERα degradation; and aromatase inhibitors (AIs), which block the conversion of androgens to estrogens^58^. Nevertheless, resistance to endocrine therapy frequently emerges, largely due to genetic and epigenetic alterations that perturb ERα signaling. A deeper understanding of novel regulatory mechanisms governing ERα is therefore essential to elucidate the basis of endocrine resistance. In the following sections, we highlight recent advances in the genomic regulation of *ESR1* itself, epigenetic co-regulation of ERα-induced transcription, and post-translational modification of ERα.

### Genomic regulation of *ESR1*

The genomic regulation of *ESR1* is fundamental to ERα expression and has profound implications for BCa progression. Alterations in *ESR1*, including mutations or dysregulated expression, can disrupt ERα function and thereby influence key biological processes such as cell proliferation or apoptosis^59–61^. These regulatory changes occur not only within tumor cells but also through interactions with signaling pathways in the surrounding microenvironment, collectively driving BCa development. As ERα is a central target of endocrine therapy, its expression status directly impacts therapeutic response. The loss of ERα expression, often reflected in the transition from ERα-positive (ERα+) to ERα-negative (ERα-) status, is widely regarded as a marker of malignant progression^16,58^. Such alterations contribute to resistance against conventional endocrine therapies, complicating clinical management^16,22^. Moreover, extensive *ESR1* promoter methylation, the presence of ERα splice variants, and recurrent ERα mutations further modulate receptor expression and function. Collectively, these genomic alterations critically shape ERα signaling and determine the efficacy of endocrine therapy^62–64^.

#### DNA methylation

DNA methylation is a key epigenetic mechanism that regulates gene expression without altering the underlying DNA sequence^65^. It involves the reversible addition of methyl groups to the fifth carbon of cytosine, catalyzed by DNA methyltransferases (DNMTs), and typically results in transcriptional silencing^66^. Aberrant DNA methylation patterns are a hallmark of BCa, occurring both genome‑wide and at specific loci^67^. Alterations in the methylation status of *ESR1*, its downstream targets, and upstream regulators profoundly influence ERα expression and endocrine therapy response^68,69^. Hypermethylation of the *ESR1* promoter has been consistently associated with poor prognosis and reduced sensitivity to endocrine therapy in both primary and metastatic BCa^62,70^. For example, IL‑1β‑induced upregulation of TWIST1 promotes *ESR1* promoter methylation, leading to decreased ERα expression and tamoxifen resistance^71,72^. Similarly, ZEB1 recruits DNMT3B and HDAC1 to the *ESR1* promoter, inducing hypermethylation and transcriptional silencing, thereby contributing to antiestrogen resistance^73^. The SNAI2-DNMT3B complex exerts a comparable effect, driving epithelial mesenchymal transition and correlating with poor outcomes in ERα‑positive tumors^70^.

Conversely, demethylation mechanisms can restore ERα expression. TET2, in cooperation with FOXP1, promotes *ESR1* demethylation, whereas TET2 loss reduces ERα levels and enhances tamoxifen resistance^74^. At the enhancer level, Stone et al. demonstrated that DNA hypermethylation at estrogen‑responsive enhancers diminishes ERα binding and suppresses the expression of key regulatory genes^75^. In addition, hypermethylation of the transcription factor SALL2 reduces ERα expression through activation of the Akt/mTOR pathway, further promoting tamoxifen resistance^76^. Clinical studies reinforce these mechanistic insights. Breast tumors from tamoxifen‑resistant patients exhibit elevated DNMT1, DNMT3A, and DNMT3B expression compared with tamoxifen‑sensitive cases. Pharmacological inhibition of DNMTs restores ERα and E‑cadherin expression, reduces invasion and metastasis, and enhances responsiveness to tamoxifen, highlighting the therapeutic potential of targeting DNA methylation in endocrine‑resistant BCa^76–78^.

#### ERα variants

Splice variants of ERα add an additional layer of complexity to estrogen signaling in BCa. Beyond the canonical full‑length ERα66, multiple isoforms generated through alternative splicing or alternative translation initiation exhibit distinct structural features, subcellular localizations, and signaling properties that profoundly influence tumor biology and therapeutic response.

ERα36, with a molecular weight of ~36 kDa, lacks both AF-1 and AF-2 but retains the DNA‑binding domain and part of the ligand‑binding domain, together with a unique C‑terminal sequence. Predominantly localized to the plasma membrane and cytoplasm^79,80^. ERα36 mediates rapid nongenomic signaling through PKC, c-Src/ERK, and EGFR/HER2 pathways, including direct interaction with ERK2 to sustain its phosphorylation, and cooperates with STAT3/p300 to enhance MMP2/9 expression and migration. It also modulates progesterone receptor signaling^79,81–84^. ERα36 is directly activated by tamoxifen, which upregulates ALDH1A1, augments stemness, and drives metastasis. Additional mechanisms include CUL4B‑mediated repression of miR-32-5p and activation of the SphK1/S1P axis^85–88^. Clinically, high ERα36 expression correlates with poor outcomes in tamoxifen‑treated ERα66‑positive patients and contributes to ERα+ to ERα- conversion, epithelial-mesenchymal transition (EMT), and therapy resistance via the 14-3-3τ/AKT/GATA3/ERα36 cascade^89,90^. Therapeutically, ERα36 represents a targetable node, as agents such as curcumol or inhibitors of the SphK1/S1P pathway suppress ERα36‑dependent signaling and tumor growth^88,91,92^.

ERα46, a 46 kDa isoform translated from an alternative start codon in exon 2, lacks the AF‑1 domain but retains the DNA- and ligand-binding domains^93–95^. ERα46 is frequently detected in breast tumors, often more abundant than ERα66 in well‑differentiated, low ERα-grade cancers, and its nuclear ratio relative to ERα66 decreases with malignant progression^94,96^. Functionally, ERα46 antagonizes ERα66 by repressing AF-1-dependent transcription and estradiol‑induced gene expression, and unliganded ERα46 maintains a repressed chromatin state through corepressor recruitment^93,97^. Loss of ERα46 is associated with tamoxifen resistance, whereas re-expression restores drug sensitivity. Splicing regulators such as HMGA1a and BARX2 modulate ERα46 levels, thereby influencing tamoxifen response, cell growth, and invasion^98,99^. Beyond endocrine therapy, ERα46 cooperates with insulin/insulin receptor signaling to promote tumor growth and metastasis, and induces IL‑11 to remodel the extracellular matrix and enhance fibroblast motility, underscoring its distinct transcriptional output and diagnostic relevance^94,96,100^.

Other splice variants, including ERαV/ΔE3-6, ERα‑LBD, and ERαΔ4, have also been identified in BCa cells. ERαV/ΔE3-6, lacking exons 3–6, suppresses wild‑type ERα transcriptional activity, though its physiological role remains unclear. In contrast, ERα‑LBD, composed mainly of the ligand‑binding domain, promotes cell growth and fulvestrant resistance but is absent in normal breast tissue. ERαΔ4, missing exon 4, has been detected in MCF‑7 cells and ovarian carcinoma, and its altered localization and signaling suggest a potential role in modulating estrogen responses and tumor progression. These variants are reviewed by Saito^21^.

Together, these ERα variants highlight the structural and functional diversity of estrogen receptor signaling in BCa. By modulating transcriptional activity, engaging non‑genomic pathways, and shaping endocrine responsiveness, they not only complicate the interpretation of ER status in tumors but also offer potential biomarkers and therapeutic targets for precision intervention.

#### ERα mutations and fusions

Multiple studies have demonstrated that ESR1 mutations predominantly cluster within the LBD, most notably at residues Y537 and D538 (e.g., Y537S, D538G). These alterations stabilize the agonist conformation of helix 12, thereby maintaining receptor transcriptional activity in the absence of ligand and conferring constitutive activation to tumor cells. The strength of ligand-independent activity varies among mutations: Y537S exhibits the most potent constitutive activation, whereas D538G enhances receptor stability by relieving conformational strain between helices 11 and 12^64,101,102^. Clinically, ESR1 mutations are exceedingly rare in primary breast tumors but occur in 20–40% of ERα+ metastatic BCa following AI therapy^63,103,104^. They represent a principal mechanism of acquired AI resistance and are associated with disease progression, enhanced metastatic potential, and poor prognosis. Among the recurrent hotspots, Y537S confers the strongest ligand-independent activity, marked endocrine resistance, and shorter survival, whereas D538G stabilizes the receptor by relieving helix 11-12 strain, is linked to liver metastasis, and may activate Wnt signaling. Y537S also imparts greater resistance to tamoxifen and fulvestrant than D538G^60,63,102,105,106^. Beyond these canonical sites, other LBD mutations such as L536 substitutions and E380Q also stabilize helix 12, producing intermediate constitutive activity and reduced sensitivity to endocrine therapies, though generally less potent than Y537S, and are enriched in AI‑treated metastases^59,60,101,107^. A broader spectrum including S463P, V534E, Y537N/C/H, and less common variants such as K303R, V392I, P535H, and V524E has been described. Most functionally resemble Y537/D538 by promoting ligand-independent ERα activity and endocrine resistance, but some display distinct mechanisms: K303R and E380Q confer estrogen hypersensitivity; S432L and V534E retain hormone-dependent activation and are considered functionally neutral; and V534E uniquely inhibits ERα SUMOylation, paradoxically enhancing ERα-mediated transcription in the presence of SERDs^106,108,109^.

Prominent *ESR1* alterations in endocrine‑resistant metastatic BCa include frequent LBD missense mutations and less common but clinically relevant *ESR1* gene fusions. LBD mutations confer constitutive receptor activity and reduced endocrine sensitivity, whereas *ESR1* fusions generate chimeric proteins that drive estrogen-independent growth and therapy resistance^110^. A recurrent rearrangement, ESR1-e2 > CCDC170, fuses the 5′ untranslated region of ESR1 to the coding region of CCDC170, producing truncated ΔCCDC170 proteins that enhance cell motility and growth. Functional studies further demonstrated that these fusions engage the HER2/HER3-Src-AKT signaling axis, thereby reducing endocrine sensitivity and contributing to therapy resistance^111,112^. Beyond the recurrent ESR1-CCDC170 fusions, additional ESR1‑e6 fusions have been identified in therapy‑refractory BCa. These retain the AF‑1 and DNA‑binding domains but lose the LBD, acquiring partner transactivation domains. ESR1‑e6 > YAP1 drives potent ligand‑independent transcriptional activity^113^, while SOX9 and DAB2 fusions similarly reprogram ERα signaling to enhance proliferation, survival, and endocrine resistance^114^, as summarized in recent reviews^115^.

Genomic alterations of ESR1, encompassing promoter hypermethylation-mediated silencing, diverse splice variants with distinct signaling properties, and ligand-binding domain mutations or fusions conferring constitutive activity, collectively perturb ERα expression and function. These changes not only drive the transition from hormone-dependent to hormone-independent growth but also fundamentally impair endocrine therapeutic efficacy, underscoring the clinical necessity of assessing ERα genomic status in treatment-resistant breast cancer.

### Epigenetic co-regulation of ERα-induced transactivation in BCa

The transcriptional programs mediated by ERα are orchestrated through the coordinated action of diverse epigenetic co-regulators that assemble into receptor-associated complexes. These factors remodel chromatin at enhancer and promoter regions adjacent to EREs, thereby either facilitating or constraining the transcription of ERα target genes^69^. Accumulating evidence highlights the pivotal role of histone post-translational modifications (PTMs) in shaping ERα signaling in BCa^14^. According to their functional activities in depositing, removing, or interpreting epigenetic marks, these regulatory proteins are generally categorized as writers, erasers, and readers^116^. Through histone acetylation, methylation, and ubiquitination, they establish chromatin states that influence a wide range of biological processes in BCa cells, including proliferation, apoptosis, invasion, and metabolic reprogramming^9,14,68,117,118^.

Histone PTMs influence ERα signaling through shared enzymatic mechanisms. The enzymes that catalyze histone acetylation, methylation, and ubiquitination, including acetyltransferases and deacetylases, methyltransferases and demethylases, as well as ubiquitin ligases and deubiquitinases, play a pivotal role in shaping chromatin accessibility and transcriptional competence. Through these activities, histone modifications establish permissive or repressive chromatin states that critically influence ERα recruitment to target loci and the transcriptional output of estrogen-responsive genes^117,119,120^. Notably, the regulatory scope of these enzymes is not restricted to histones. Many of them also act on non-histone substrates, including ERα itself, thereby extending their influence from chromatin remodeling to direct receptor regulation. This enzymatic overlap highlights a unifying regulatory logic: the same catalytic systems that remodel nucleosomes also fine‑tune ERα stability, localization, and signaling capacity. While the chromatin-centered effects are discussed here, their receptor-centered actions will be elaborated in Section (III).

#### Histone acetylation

Histone acetylation is catalyzed by histone acetyltransferases (HATs), which transfer an acetyl group from acetyl-CoA to the ε-amino group of lysine residues, generally leading to chromatin relaxation and transcriptional activation^119,121^. For example, acetyltransferases KAT6A and KAT6B upregulate estrogen signaling genes by increasing histone H3K23Ac levels, thereby influencing treatment outcomes in refractory BCa endocrine therapy^122^. Histone acetyltransferases CBP/p300 markedly increase ERα-mediated gene transcription^123^, while alterations or deficiencies in activation 3 (ADA3) facilitate the recruitment of p300, PCAF (KAT2B), and GCN5 (KAT2A) to EREs, promoting estrogen-dependent BCa cell proliferation^124^.

Conversely, histone deacetylases (HDACs) counteract these modifications. In the presence of Tamoxifen, increased recruitment of HDAC1 and HDAC2 at the downstream promoter region of ERα reduces histone acetylation and inhibits ERα-mediated transcription of FOXM1^56^. ARID1A recruits SWI/SNF complex and HDAC1 to ERE, diminishing histone H4 acetylation and inhibiting downstream target gene transcription^125,126^. Similarly, the co-repressor TLE3 recruits HDAC2 to suppress a subset of ERα target genes^127^. Notably, HDAC3 cooperates with ERα to occupy the p53-binding site on the CDKN1A (p21) promoter, repressing its expression^128^. Additionally, SIRT1 is recruited by ERα to transcriptionally suppress the TP53 gene, thereby attenuating the P53-mediated apoptotic response^129^. Clinically, Phase II trials demonstrate that exemestane combined with entinostat improves progression-free survival (PFS) and overall survival (OS) in aromatase inhibitors (AI) -resistant patients^130^, while the selective HDAC inhibitor tucidinostat enhances PFS in endocrine-resistant ERα+BCa^131^.

Collectively, histone acetylation represents the most clinically advanced epigenetic layer in endocrine-resistant breast cancer, with a relatively clear functional dichotomy between HAT-mediated activation and HDAC-mediated repression. The existence of FDA-approved HDAC inhibitors and ongoing Phase II clinical trials (e.g., entinostat, tucidinostat) underscores the translational maturity of this field. Nevertheless, isoform-selective inhibition remains a significant challenge: pan-HDAC inhibitors may interfere with non-ERα-related cellular processes, while compensatory activation of specific HDAC family members (e.g., HDAC3/SIRT1) can compromise therapeutic efficacy. Future investigations should focus on distinguishing HDAC complexes specifically recruited to ERα-bound loci from those acting within global chromatin environments, thereby minimizing off-target effects.

#### Histone methylation

Histone methylation on lysine and arginine residues is deposited by lysine methyltransferases (KMTs), and protein arginine methyltransferases (PRMTs) and removed by lysine-specific demethylases (KDMs) and peptidylarginine deiminases (PADs), respectively, generating marks with site- and state-specific outcomes for transcription^12,132^. Silencing-associated methylation (e.g., H3K9, H3K20, H3K27) and activating marks (e.g., H3K4, H3K36, H3K79, H3R2, H4R3) shape the accessibility and co-regulator landscape at ERα-bound regions, thereby tuning estrogen-dependent transcriptional programs. The precise spatiotemporal control of methylation is essential for epigenomic homeostasis and is linked to tumor progression, therapeutic resistance, invasiveness, and microenvironmental remodeling^133,134^.

To date, a number of enzymes that control methylation status of histone have been identified as co-regulators of ERα signaling. H3K4 methyltransferase SETD1A is expressed at elevated levels and is significantly correlated with a lower survival rate in patients with ERα+ tamoxifen-resistant BCa. SETD1A facilitates the recruitment of ERα and establishes accessible chromatin at enhancer regions of ERα target genes enriched in cell cycle and cancer-promoting pathways^135,136^. Recent studies demonstrate that the pioneer factor FOXA1 recruits the H3K4 methyltransferase KMT2C (also known as MLL3) to ERα-bound enhancers, where KMT2C deposits H3K4me1 to demarcate active enhancer landscapes essential for receptor-mediated transcription^137^. Consequently, KMT2C knockdown selectively reduces both H3K4me1 and H3K27ac at these enhancers, impairing estrogen-dependent gene expression and proliferation. Notably, while KMT2C loss suppresses hormone-driven growth, it paradoxically promotes tumor outgrowth under hormone-depleted conditions, highlighting its context-dependent role in breast cancer progression^138^. PLK1 interacts with ERα and cooperatively recruits to the ERE region, where it enhances the activity of methyltransferase KMT2B to increase the level of histone H3K4me3, thereby promoting the transcription of downstream ERα target genes^139^. The histone H3K9 methyltransferase G9a is recruited to both the *TFF1* and *PGR* promoters, yet it exerts opposing regulatory effects, being indispensable for estrogen‑induced *TFF1* activation while repressing *PGR* transcription. By contrast, KMT2A (MLL1) is uniformly required for estrogen‑driven expression of both genes and enhances ERα occupancy at their regulatory regions^140^. In addition, Histone H3K27 methyltransferase EZH2 has been demonstrated to play a crucial role in numerous tumors, and pharmacologic inhibition of EZH2 enhances therapeutic efficacy against tamoxifen-resistant BCa^141^. As a histone H3K36 methyltransferase, NSD2 (also known as WHSC1) is specifically recruited to the ESR1 gene locus via BET proteins BRD3/4-mediated recognition of acetylated histone tails. Once localized, it catalyzes H3K36me2/3 to facilitate transcriptional elongation and maintain high-level ERα expression, forming a self-reinforcing positive feedback loop wherein estrogen signaling upregulates NSD2, which in turn promotes ERα transcription. Concurrently, NSD2 functions as an ERα coactivator, synergistically enhancing receptor-mediated transactivation of downstream target genes. The small-molecule BET inhibitor JQ1 disrupts the bromodomain-mediated interaction between BRD3/4 and acetylated histones, thereby simultaneously abrogating both regulatory mechanisms, reducing ERα protein abundance and impairing its transcriptional activity, which confers potent antitumor efficacy in tamoxifen-resistant breast cancer models and produces synergistic suppression when combined with the ERα degrader fulvestrant^142^. Separately, DZNep induces proteasomal degradation of NSD2 protein, triggering apoptosis and inhibiting tumor growth in tamoxifen-resistant breast cancer cells^143^. Methylation of histone H4 at arginine 3 (H4R3) facilitating transcriptional activation by ERα^144^. It has been reported that PRMT1 catalyzes the methylation of histone H4R3 and upregulates ERα-mediated *TFF1* transcription in BCa cells^145^. CARM1, also known as PRMT4, enhances the methylation of Histone H3 at residues R2, R17, and R26 to promote gene activation induced by ERα^146–149^.

Conversely, histone demethylases function as complementary epigenetic regulators that reverse methylation marks to dynamically modulate ERα transcriptional activity. The histone demethylase KDM3A, as a positive regulator of ERα activity, enhances the recruitment of ERα to EREs within target gene promoters by demethylating histone H3K9, which is essential for growth in a model of endocrine therapy-resistant BCa^150^. A regulatory relationship exists among demethylases, with evidence indicating that KDM4B can enhance the expression of KDM3A. Furthermore, KDM3A recruits KDM4B and FOXA1 to EREs by reducing the levels of histone H3K9me1/me2, creating an appropriate environment for subsequent recruitment of transactivating factors, such as BRD4 and p300, further upregulating ERα-mediated gene transcription and promoting the growth of BCa^151^. Interestingly, the demethylase KDM4B and methyltransferase KMT2B form a complex that catalyzes the demethylation of histone H3K9 and the methylation of histone H3K4 on downstream target genes mediated by ERα, thereby enhancing the transcriptional activity of ERα and promoting the G1/S transition in the cell cycle as well as breast tumorigenesis^152^. Histone lysine demethylase KDM6A collaborates with acetyltransferase CBP to regulate the transition between H3K27 methylation and acetylation, thereby establishing a permissive chromatin environment that enhances ERα-induced gene transcription. Furthermore, upon estrogen stimulation, ERα transactivates KDM6A, creating a feed-forward loop that facilitates BCa progression^153^. Inhibition of KDM5A/B enhances sensitivity to anti-estrogens treatment by modulating ERα signaling and reducing cellular transcriptomic heterogeneity in BCa exhibiting endocrine resistance^154,155^. As a dual-specificity demethylase targeting both H3K4 and H3K9, LSD1 (KDM1) exhibits versatile roles in ERα-mediated transcriptional regulation, capable of either promoting or repressing gene expression depending on cofactor assembly and substrate specificity. Upon recruitment by ERα and the reader protein PELP1, LSD1 switches its substrate preference from H3K4me2 to H3K9me2, thereby erasing repressive marks to facilitate transcriptional activation^156^. Furthermore, within the KMT2B-LSD1-KDM6A complex recruited to ERE regions, LSD1 contributes to the coordinated removal of repressive H3K9me2/H3K27me3 marks alongside establishment of activating H3K4me3, promoting target gene expression^157^. Conversely, under alternative contexts, LSD1 can function as a transcriptional repressor independent of its demethylase activity, increasing H3K9me2 and reducing H3K9Ac to promote BCa cell proliferation and endocrine resistance^158^.

Unlike the binary regulatory logic of acetylation, histone methylation constitutes a more intricate regulatory network. Its complexity stems from the diversity of modification sites with distinct, site-specific transcriptional outcomes and from the context-dependent multifunctionality of modifying enzymes. Individual enzymes (e.g., LSD1, KDM4B, G9a) often possess both catalytic and scaffolding functions, capable of differentially modulating ERα-mediated gene expression depending on the specific genomic loci they occupy. This combinatorial complexity, together with synergistic multi-enzyme complex assembly (e.g., KMT2B-LSD1-KDM6A), creates a highly elaborate regulatory landscape. Therapeutically, in addition to direct enzymatic inhibition, indirect modulation through disruption of transcriptional complex assembly (such as indirectly targeting NSD2) also demonstrates therapeutic potential. However, given the site-specific nature of methylation marks and the multifunctionality of associated enzymes, developing targeted interventions remains challenging, necessitating further elucidation of precise enzyme-substrate mechanisms in ERα signaling.

#### Histone ubiquitination

Monoubiquitination of H2A (H2Aub1, Lys-119) and H2B (H2Bub1, Lys 120/123) is installed via the canonical E1-E2-E3 cascade and produces context-dependent transcriptional outcomes. H2Aub1 typically represses gene expression, whereas H2Bub1 can either promote or constrain transcription depending on locus-specific distribution of modifying complexes and cofactor interactions^159,160^. Although studies focused on ERα-driven transcription remain limited, emerging evidence implicates histone ubiquitination as a significant modulator of ERα+BCa, influencing enhancer function and signal integration.

H2Bub1 catalyzed by the RNF20/RNF40 E3 ligase complex, exerts subtype‑specific and context-dependent effects in breast cancer. In luminal subtypes, RNF20 driven H2Bub1 promotes transcriptional elongation of ERα target genes such as *PGR*, *CXCL12*, and *FOXA1*, thereby reinforcing estrogen receptor signaling; conversely, the deubiquitinase USP44 antagonizes this modification, producing effects opposite to RNF20. In basal‑like subtypes, however, RNF20-mediated H2Bub1 acts in a tumor-suppressive manner by repressing NF-κB-driven pro-inflammatory cytokine expression^161^. At enhancer regions, H2Bub1 also restrains ERα enhancer activation by stabilizing the histone variant H2A.Z and preventing its eviction by the INO80 remodeler^162^. RNF40, together with RNF20, is required for H2Bub1 dependent transcriptional elongation of ERα target genes. Loss of RNF40 reduces ERα-driven transcription, but at the same time triggers compensatory activation of AKT and ERK signaling pathways, which can sustain estrogen‑independent proliferation of breast cancer cells^163^. Beyond RNF20/RNF40, other ubiquitin ligases also intersect with ERα regulation: the CUL4B‑RING complex represses miR‑32‑5p via H2Aub1, thereby relieving its inhibition of ERα36 and contributing to tamoxifen resistance^87^; and the Polycomb protein RNF2 (RING1B) enhances ERα mediated transcription by promoting enhancer-promoter interactions and R-loop formation, independently of its canonical H2Aub1 activity^164^. Collectively, these findings highlight the multifaceted and sometimes opposing roles of histone ubiquitination in shaping ERα driven transcriptional programs and breast cancer pathogenesis. The human Suppressor of Ty Homologue-6 (SUPT6H) functions as a histone chaperone that facilitates the ubiquitination of histone H2B and diminishes levels of histone H3K27me3, thereby promoting ERα-mediated transcription of genes associated with cell differentiation, potentially through the recruitment of KDM6 and RNF40^165^.

Our early work indicated that the TFTC/STAGA complex can co-activate nuclear receptors by removing both H2Aub1 and H2Bub1, thereby counteracting heterochromatin silencing^166^. Subsequent studies revealed that the SAGA-module subunit ATXN7L3 facilitates the deubiquitylation of histone H2Bub1 and promotes the methylation of histone H3K4, thereby enhancing ERα target gene expression, despite lacking intrinsic catalytic activity^167^. More directly, our recent work demonstrated that the deubiquitinase MYSM1 enhances ERα-mediated transactivation by erasing H2Aub1 while simultaneously promoting the accumulation of active chromatin marks, including H3K4me3, H3K9Ac and H3K27Ac^168^.

Compared with acetylation and methylation, the investigation of histone ubiquitination in ERα transcriptional regulation remains in its early stages, with existing evidence revealing its marked functional duality and extensive crosstalk with other epigenetic marks. H2B monoubiquitination exhibits subtype-specific effects, promoting transcriptional elongation of ERα target genes in luminal cells while suppressing NF-κB-driven pro-inflammatory pathways in basal-like subtypes, whereas its removal paradoxically correlates with active transcription through coupling with H3K4 methylation. Notably, current research on E3 ubiquitin ligases in estrogen receptor signaling has focused predominantly on modulating receptor protein stability, while specific enzymes mediating histone ubiquitination and their molecular mechanisms remain to be systematically characterized. It is noteworthy that E3 ubiquitin ligases may also participate directly in chromatin architectural regulation through non-enzymatic mechanisms, such as facilitating enhancer-promoter interactions and R-loop formation; these catalytic activity-independent structural functions require refined mechanistic dissection. Furthermore, compensatory activation of kinase signaling (AKT/ERK) upon disruption of H2Bub1 elongation complexes suggests that interventions targeting histone ubiquitination may require combinatorial strategies to overcome rapid adaptation.

#### Other co-regulators of ERα

**Fig. 2:**
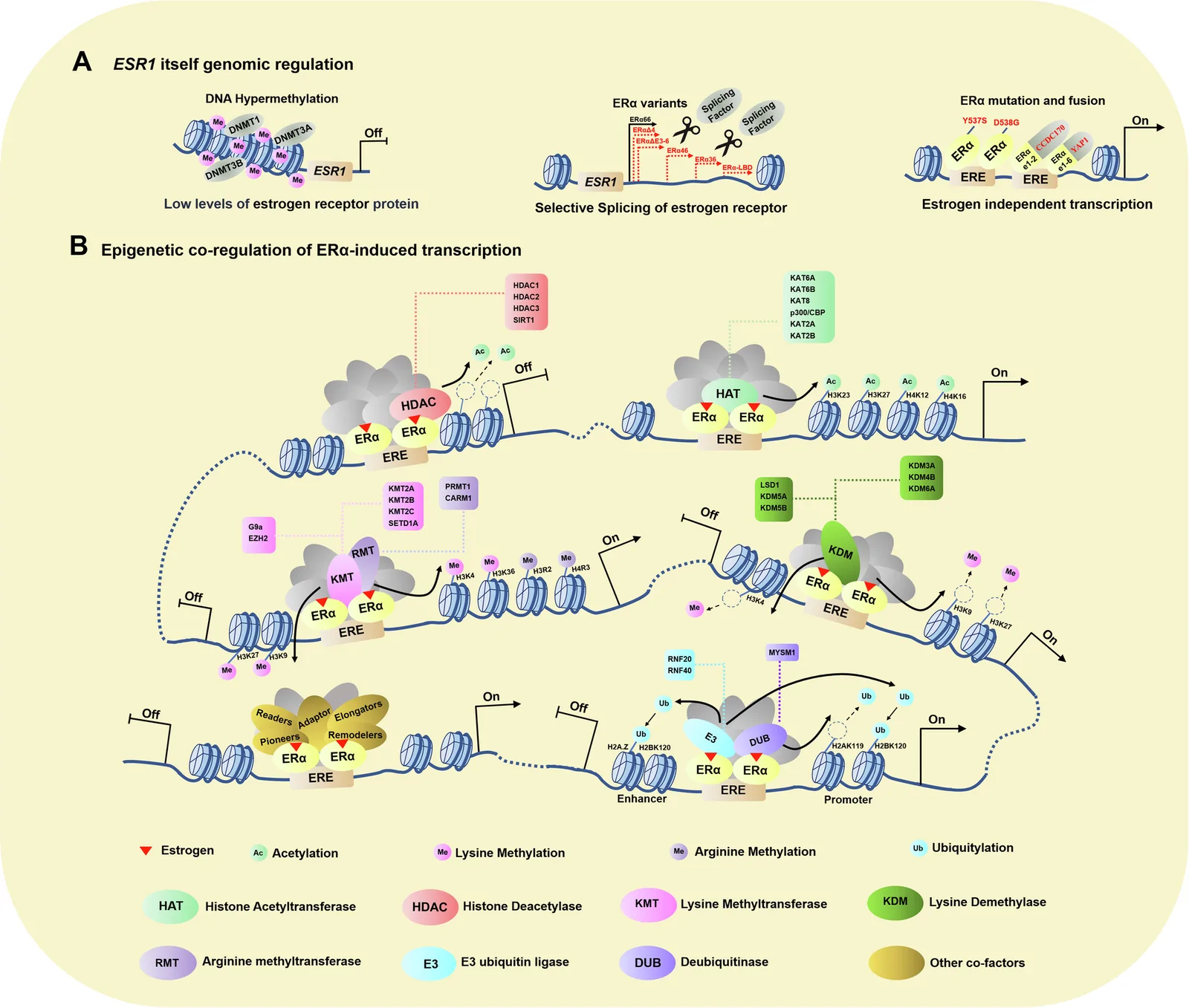
A schematic illustration for genomic and epigenomic regulation of the ERα signaling pathway in Breast Cancer. **A** *ESR1* is genomically regulated through DNA methylation, mRNA alternative splicing, point mutations, and gene fusions with other transcription factors; **B** Epigenetic co‑regulation of ERα‑mediated transcription involves histone acylation, methylation, and ubiquitination. Colored boxes denote individual proteins, which are connected by color‑matched dashed lines to an oval indicating their collective functional category. For histone modifications illustrated here (methylation, acetylation, and ubiquitination), the enzymes are positioned in closest proximity to the corresponding modification sites to clearly indicate their site‑specific regulatory roles. The red tri-angle indicates estrogen. DNMT DNA methyltransferase, ERα66 full‑length isoform, ERαΔ4 exon‑4 deleted isoform, ERαΔ3‑6 exons 3-6 deleted isoform, ERα46 AF‑1 truncated isoform, ERα36 AF‑1/AF‑2 truncated, membrane‑associated isoform, ERα-LBD ligand‑binding‑domain-only isoform. ERα Y537S/D538G ligand‑binding domain mutations, ERα‑CCDC170 (e1‑2) N‑terminal fusion protein, ERα‑YAP1 (e1‑6) N‑terminal fusion protein, ERE estrogen response element, Me methylation, Ac acetylation, Ub ubiquitination, HAT histone acetyltransferase, HDAC histone deacetylase, KMT lysine methyltransferase, RMT protein arginine methyltransferase, KDM lysine demethylase, E3 E3 ubiquitin ligase, DUB deubiquitinase. Pioneers, proteins that open closed chromatin (FOXA1, AP2γ); Readers, proteins that recognize epigenetic marks (BRD4, BRPF1); Remodelers, proteins that reposition or restructure nucleosomes (SWI/SNF subunits BRG1, BAF57); Elongators, proteins that promote transcriptional elongation (RAC1); Adapters, proteins that bridge transcription factors and epigenetic-modifying enzymes (SRC‑1/2/3, HOXB7, BAP18, MDC1, N‑CoR, SMRT).

Other ERα transcriptional co-regulators, ranging from chromatin remodelers to pioneer and architectural factors, influence receptor signaling by either promoting or repressing transcription. The p160 Steroid Receptor Coactivator (SRC) family, comprising SRC-1, SRC-2, and SRC-3, functions as critical coactivators of sexual hormone receptors. Phosphorylated SRC2 enhances its interaction with CBP/p300, thereby strengthening coactivation of ERα, PR, and AR. Similarly, estrogen-induced phosphorylation of SRC-3 at Y1357 by c-Abl promotes binding to p300 and supports ERα- and PR-dependent transcription^169^. In addition, MUC1 associates with ERα and facilitates recruitment of SRC-1/2, leading to increased ERα transcriptional activity and hormone-dependent proliferation of breast cancer cells^170^. SWI/SNF remodeling sub-units BRG1 and BAF57 further potentiate this response by supplying ATP-dependent nucleosome sliding that increases ERα DNA accessibility^9^. Pioneer factor usage switches during early epithelial-mesenchymal transition: FOXA1 dominates in non-invasive disease to preload ERα at proliferation genes, whereas AP2γ displaces FOXA1 and directs ERα into a ZEB1-containing complex that activates invasion and metastasis genes^171^. HOXB7 acts as an ERα co-factor on the HER2 promoter and other targets to promote endocrine resistance^172^. BRD4 (an epigenetic reader) facilitates ERα to stabilize enhancer-promoter loops that drive oncogenes^173^, and RAC1 sustains RNA-Pol-II elongation at both promoters and enhancers^174^. The effectiveness of endocrine therapy can be enhanced by downregulating the ERα binding protein BRPF1, which regulates the accessibility of ERα to chromatin^175^. Our previous studies demonstrate that BAP18 potentiates ERα signaling through dual mechanisms: it recruits the COMPASS complex to catalyze H3K4 methylation at target loci and cooperates with CTCF to establish promoter-enhancer chromatin looping, further amplifying ERα signaling pathway^176–178^. Conversely, MDC1 can act as a context-dependent co-activator at EREs to induce p21, ultimately inhibiting BCa growth^179^. Nuclear receptor corepressors N-CoR and SMRT, in complex with Sin3 and HDACs, are selectively recruited by antagonist-bound ERα to repress transcription, thereby mediating the inhibitory action of tamoxifen; notably, reduced N-CoR levels correlate with acquired tamoxifen resistance, underscoring the critical balance between coactivator and corepressor complexes in determining ERα signaling outcomes^180^ (Fig. 2).

In summary, the chromatin-centric modifications of acetylation, methylation, and ubiquitination establish a context-dependent epigenetic framework governing ERα transcription with remarkable functional plasticity. These mechanisms underscore the complexity of therapeutic targeting in endocrine-resistant disease. Extending beyond histone remodeling, this regulatory paradigm applies to direct post-translational modifications of the ERα protein itself, which diversify receptor activity through mechanisms independent of chromatin marks and are elaborated in the following section.

### Post-translational modification of ERα protein in BCa

Beyond remodeling chromatin architecture, many enzymes that catalyze histone modifications also target non-histone substrates. ERα represents a prominent example of this functional convergence; numerous histone acetyltransferases, methyltransferases, E3 ligases, and deubiquitinases have been shown to modify the receptor. These post-translational modifications establish an independent regulatory layer that encodes ERα functional states, controlling protein stability, subcellular trafficking, DNA binding affinity, and cofactor interactions, thereby diversifying transcriptional output beyond epigenetic templating. Importantly, these marks do not operate independently. Distinct modifications at specific residues exhibit cross-talk, wherein one PTM can enhance or antagonize another, creating a combinatorial code that fine-tunes receptor activity^15,181–186^.

**Fig. 3:**
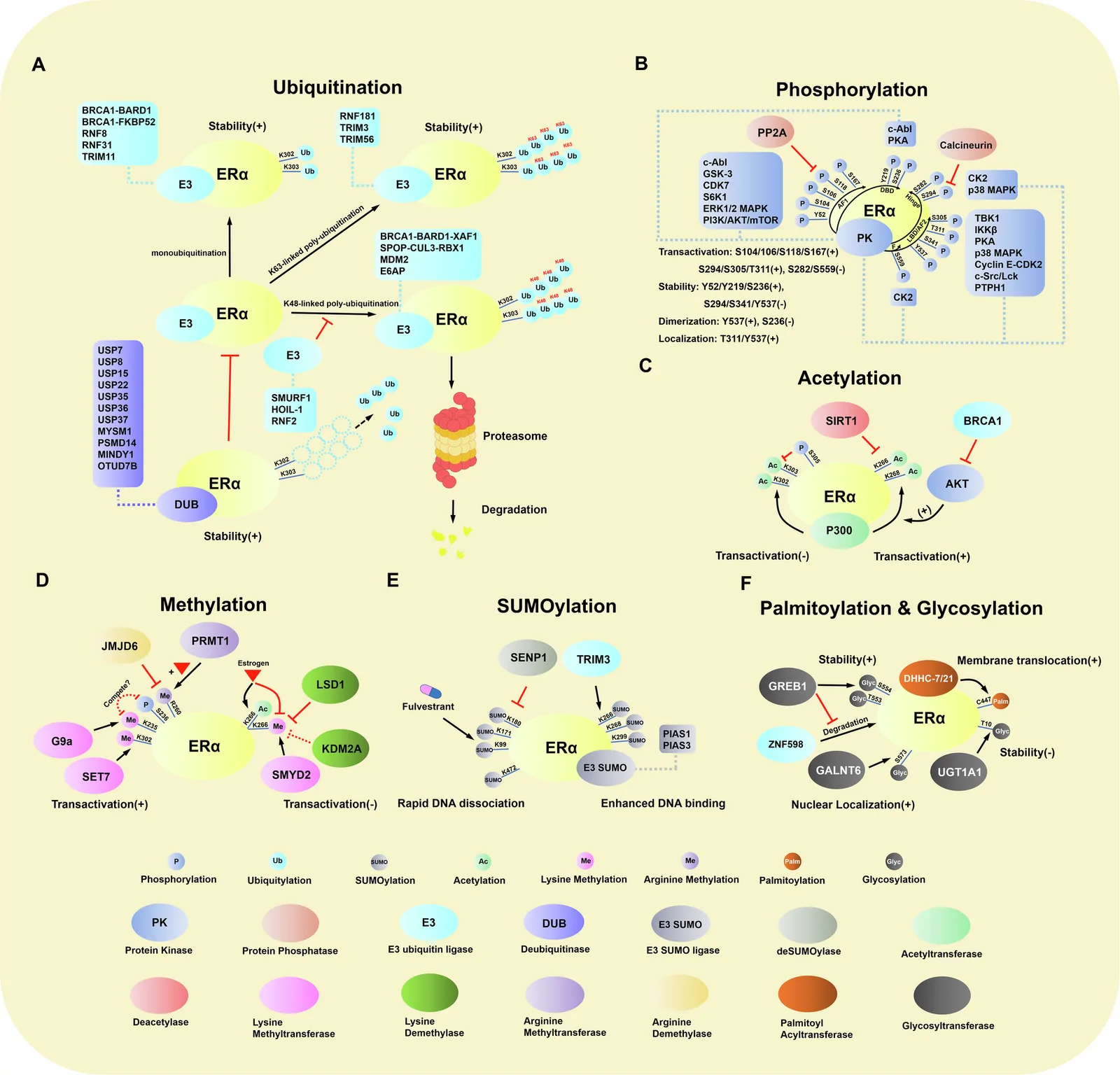
A schematic overview of post‑translational modifications of ERα in breast cancer. **A** ERα ubiquitination, including monoubiquitination and K48‑ or K63‑linked polyubiquitination, together with the relevant E3 ligases and deubiquitinases (DUB). Monoubiquitination and K63‑linked polyubiquitination enhance protein stability, whereas K48‑linked polyubiquitination promotes proteasomal degradation. **B** ERα phosphorylation sites with corresponding protein kinases (PK) and phosphatases. Phosphorylation of ERα at specific sites regulates transcriptional activity, protein stability, dimerization, and nuclear localization; (+) denotes promotion, (−) denotes inhibition. **C**, **D** ERα acetylation and methylation sites with the relevant acetyltransferases/deacetylases and methyltransferases/demethylases. Acetylation or methylation at different residues may either promote (+) or inhibit (−) ERα transcriptional activity, and these two modifications can compete for occupancy at the same site. The red tri-angle indicates estrogen. **E** ERα SUMOylation, including modification sites together with the relevant SUMO E3 ligases and deSUMOylating enzymes. SUMOylation of ERα modulates DNA binding and chromatin interaction, thereby influencing transcriptional regulation. **F** ERα palmitoylation and glycosylation, together with the relevant modifying enzymes. These modifications alter ERα protein stability and regulate its subcellular distribution, including nuclear localization and membrane association. Colored boxes denote individual proteins, which are connected by color‑matched dashed lines to an oval indicating their collective functional category. For modifications with multiple sites, such as phosphorylation, the enzymes catalyzing specific residues are positioned in closest proximity to the corresponding modification sites for clarity. AF-1 Activation Function-1 domain, DBD DNA-Binding Domain, LBD/AF-2 Ligand-Binding Domain/Activation Function-2, P phosphorylation, PK protein Kinase, Ub ubiquitination, E3 E3 ubiquitin ligase, DUB deubiquitinase, SUMO SUMOylation, E3 SUMO E3 SUMO ligase, Ac acetylation, Me methylation, Glyc Glycosylation, Palm Palmitoylation.

Here, we systematically delineate how individual modification types, including ubiquitination, phosphorylation, acetylation, methylation, and SUMOylation, and emerging marks such as palmitoylation and glycosylation, serve as molecular switches to dynamically reprogram ERα activity (Fig. 3). We identify the specific modifying enzymes, residue-specific effects, and functional consequences for estrogen-dependent and independent transcription, breast cancer cellular phenotypes, and the evolution of endocrine resistance. By integrating these receptor-centric mechanisms with the chromatin-centered framework introduced earlier, we elucidate how histone and ERα modifications constitute an interconnected, bidirectional regulatory network that coordinates estrogen signaling with remarkable plasticity.

#### Ubiquitination

Accumulating evidence demonstrates that distinct ubiquitin modification patterns regulate key functional properties of ERα, including protein stability, transcriptional activity, and subcellular localization (Fig. 3A), thereby influencing the efficacy of endocrine therapy^183^. Similar to histone H2A/H2B, ERα undergoes ubiquitination via a three-step enzymatic cascade mediated by E1 activating enzymes, E2 conjugating enzymes, and E3 ligases^187^. While histones are predominantly monoubiquitinated, ERα exhibits a broader repertoire encompassing both monoubiquitination and polyubiquitination through distinct lysine linkages. Notably, K48-linked polyubiquitination generally targets ERα for proteasomal degradation, whereas K63-linked chains or monoubiquitination at residues such as K302 and K303 often enhance receptor stability or modulate its transcriptional activity^183^. The regulation of ERα by E3 ligases is remarkably complex and context dependent; members of the RING (really interesting new gene), HECT (homologous to E6-AP C-terminus), and RBR (RING-between-RING) families can either promote degradation or enhance stability, contingent upon the specific chain topology and the presence of cofactors^188–190^. This covalent marking is dynamically opposed by deubiquitinating enzymes (DUBs), which remove ubiquitin to fine-tune receptor turnover^191^. The following sections systematically delineate how specific E3 ligases and DUBs, acting through varied chain linkages and site-specific modifications, establish a sophisticated regulatory network that governs ERα signaling and therapeutic response in breast cancer.

Multiple E3 ubiquitin ligases and ubiquitin-related factors modulate ERα turnover through the ubiquitin-proteasome pathway, thereby altering receptor stability and transcriptional activity. However, these enzymes do not represent a pool of functionally equivalent catalysts; instead, they exemplify mechanistic diversification, with convergent outcomes of degradation or stabilization achieved through distinct ubiquitin chain types and protein domain targeting. Several degradation promoting enzymes, including the BRCA1-BARD1-XAF1 complex, the SPOP/CUL3/RBX1 complex, the estrogen induced transcription factor YB 1, and the MYST domain containing acetyltransferase HBO1, converge on K48 linked polyubiquitination to terminate ERα signaling^189,192–195^. In contrast, their stabilizing counterparts deploy at least three distinct biochemical strategies: direct suppression of K48 linked chains (SMURF1, HOIL-1, and RNF2)^190,196,197^, coupled monoubiquitination (RNF8, RNF31, and TRIM11)^198–200^, and K63 linked polyubiquitination (RNF181, TRIM3, and TRIM56)^201–203^. Notably, residues L429 and A430 within ERα AF-2 domain constitute a non-covalent ubiquitin binding surface that preferentially engages K63 linked chains, serving as a prerequisite for coupled monoubiquitination and subsequent stabilization^204^, whereas the FKBP52/BRCA1 complex promotes protective monoubiquitination that is competitively antagonized by FKBP51^205^. Distinct from these ubiquitin-mediated mechanisms, TRIM24 functions as a chromatin-associated co-activator. Through its tandem PHD-Bromodomain module, TRIM24 combinatorically recognizes unmodified H3K4 and acetylated H3K23, stabilizing ERα-chromatin interactions and promoting estrogen-dependent gene activation. This epigenetically guided recruitment contributes to breast epithelial proliferation and tumorigenesis^206^. These distinct topologies and domain specific engagements likely confer differential kinetic stability, subcellular trafficking, and cofactor recruitment capacities, indicating that the apparent functional overlap among ERα stabilizers or destabilizers reflects mechanistic diversification rather than simple duplication.

Beyond unidirectional degradation, a functionally distinct subset of E3 ligases couple proteasome-mediated turnover to transcriptional activation. MDM2 and E6-AP serve as dual-role co-activators that simultaneously drive target gene expression and promote ERα degradation^207–210^. This co-activator-associated protein turnover may be associated with activation-coupled proteolysis, a process in which proteasome-mediated clearance of ligand-activated receptor complexes from promoters may allow subsequent co-activator recruitment and transcriptional cycling^211,212^. Such dynamic receptor turnover potentially enables continuous estrogen sensing and prevents prolonged promoter occupancy that could dampen transcriptional responsiveness. Therefore, MDM2 and E6-AP may operate not merely as destabilizing enzymes but as components that link receptor turnover to sustained estrogen signaling output.

The dynamic balance of ERα ubiquitination is maintained by deubiquitinating enzymes that oppose E3 ligase activity. While numerous USP family members including USP7, USP8, USP15, USP35, and USP37 broadly contribute to ERα stabilization and breast cancer progression^213–218^. Other DUBs, including MINDY1 and OTUD7B, exert similar stabilizing effects^219,220^. However, these convergent stabilizing effects reflect mechanistic diversification, as individual DUBs additionally enhance ERα transcriptional activity through distinct non-proteolytic mechanisms, as evidenced by luciferase reporter assays^213–215,219,220^. This illustrates that DUB-mediated control of ERα encompasses both protein stabilization and transcriptional activation. Consistent with this dual function, pharmacological inhibition of USP14 and UCHL5 by b-AP15 and platinum pyrithione (PtPT) promotes ERα ubiquitination and suppresses its transcriptional activity, thereby inhibiting breast cancer cell proliferation in vitro^218^. In contrast, USP8 and USP35 appear to function primarily in ERα stabilization, and currently no evidence indicates that they modulate receptor transcriptional activity^216,217^. USP36 and PSMD14 have been demonstrated that each could suppress K48-linked polyubiquitination and stabilize the tamoxifen-resistant Y537S ERα mutant, with their inhibition restoring antiestrogen sensitivity and highlighting their potential as targets in resistant breast cancer^182,221^. USP11 is upregulated by estrogen signaling and functions as an ERα co-activator through a mechanism that appears independent of direct effects on receptor stability^222^. Our previous studies have further demonstrated that USP22 enhances the oncogenic output of ERα signaling by elevating both receptor activity and stability. Additionally, USP22 could also maintain HIF-1α expression, suggesting its oncogenic role under hypoxia. Down-regulating USP22 has been shown to improve endocrine therapy sensitivity in endocrine resistance cells^223^. In parallel, our recent work revealed that MYSM1 not only preserves ERα stability but also acts on chromatin by reducing histone H2A ubiquitination and influencing other histone modifications, thereby regulating ERα target genes and endocrine therapy sensitivity through both histone and non-histone substrate modification^168^. Collectively, these findings indicate that DUBs constitute a functionally diverse group in which individual members contribute to ERα regulation through distinct mechanisms, including transcriptional co-activation, mutant-specific rescue, and dual control of histone and receptor modifications, rather than serving merely as redundant stabilizers of receptor abundance (Table 1).

**Table 1:** Post-translational modifications of ERα: modifying enzymes, sites, and functional outcomes

Name	Enzyme type (complex)	Post-modification	Modification sites	Stability	Transcriptional activity
c-Abl	Tyrosine kinase	Phosphorylation	Y52 (AF-1); Y219 (DBD)	Positive	Positive
ERK1/2 MAPK	Ser/Thr kinase	Phosphorylation	S104 (AF-1); S106 (AF-1)	Positive	Positive
CDK7, ERK1/2, GSK-3	Ser/Thr kinase	Phosphorylation	S118 (AF-1)	Positive	Positive
PKA	Ser/Thr kinase	Phosphorylation	S236 (DBD); S305 (AF-2)	Positive	S236: Negative; S305: Positive
CK2	Ser/Thr kinase	Phosphorylation	S282 (Hinge); S559 (F domain)	N/A	Negative
p38 MAPK	Ser/Thr kinase	Phosphorylation	S294 (Hinge); T311 (AF-2)	N/A	Positive
TBK1, IKKβ	Ser/Thr kinase	Phosphorylation	S305 (AF-2)	N/A	Positive
c-Src, Lck	Tyrosine kinase	Phosphorylation	Y537 (AF-2)	N/A	Positive
Cyclin E-CDK2	Kinase complex	Phosphorylation	S341 (AF-2)	Negative	N/A
PI3K/AKT/mTOR/ S6K1	Kinase cascade	Phosphorylation	S167 (AF-1)	N/A	Positive
PP2A	Ser/Thr phosphatase	Dephosphorylation	S118 (AF-1)	N/A	Negative
Calcineurin	Ser/Thr phosphatase	Dephosphorylation	S294 (Hinge)	Positive	Positive (Indirectly)
PTPH1	Tyrosine phosphatase	Dephosphorylation	Y537 (AF-2)	Negative	N/A
MDM2, E6-AP	E3 ligase	Ubiquitination	N/A	Negative	Positive
SPOP-CUL3-RBX1	E3 ligase	Ubiquitination	N/A	Negative	N/A
BRCA1-BARD1-XAF1	E3 ligase complex	K48-linked poly-ubiquitination	N/A	Negative	N/A
HBO1 (MYST domain)	HAT (intrinsic E3 ligase activity)	K48-linked poly-ubiquitination	N/A	Negative	N/A
RNF8, RNF31, TRIM11	E3 ligase	Mono- ubiquitination	N/A	Positive	Positive
TRIM56	E3 ligase	K63-linked poly-ubiquitination	N/A	Positive	Positive
RNF181, TRIM3	E3 ligase	K63-linked poly-ubiquitination	N/A	Positive	Positive
SMURF1	E3 ligase	suppresses K48-linked poly-ubiquitination	N/A	Positive	Positive
RNF2, HOIL-1	E3 ligase	suppresses K48-linked poly-ubiquitination	N/A	Positive	Positive
USP7, USP15, USP22, USP36, USP37, MYSM1, PSMD14, MINDY1, OTUD7B	DUBs	Deubiquitination	N/A	Positive	Positive
USP8/35	DUBs	Deubiquitination	N/A	Positive	N/A
p300/CBP	HAT	Acetylation	K302/303 (Hinge/AF-2); K266, K268 (Hinge)	Positive	K302/303: Negative; K266/268: Positive
SIRT1	HDAC	Deacetylation	N/A	N/A	Negative
SET7	KMT	Methylation	K302 (Hinge)	Positive	Positive
G9a	KMT	Methylation	K235 (DBD)	N/A	Positive
SMYD2	KMT	Methylation	K266 (Hinge)	N/A	Negative (inhibits acetylation)
LSD1	KDM	Demethylation	K266 (Hinge)	N/A	Positive
KDM2A	KDM	Demethylation	N/A	N/A	Positive (enhance phosphorylation)
PRMT1	Arginine methyltransferase	Methylation	R260 (DBD)	N/A	Positive (enhance kinase signaling)
JMJD6	Arginine demethylase	Demethylation	R260 (DBD)	N/A	Positive (enhancer-promoter loop)
PIAS1/PIAS3	E3 SUMO ligase	SUMOylation	K266, K268 (Hinge)	N/A	Positive
SENP1	DeSUMOylase	DeSUMOylation	K171, K180 (AF-1)	N/A	Positive
DHHC-7/DHHC-21	Palmitoyltransferase	Palmitoylation	C447 (AF-2)	N/A	Positive (enhance kinase signaling)
UGT1A1	atypical UDP-GlcNAc- glycosyltransferase	UDP‑GlcNAc glycosylation (initial)	S10 (AF-1)	Negative	N/A
GREB1	O-GlcNAc transferase	O‑GlcNAcylation	T553 (AF-2) S554 (F domain)	Positive	N/A
GALNT6	O-GalNAc transferase	O‑GalNAc glycosylation	S573 (F domain)	Positive	Positive

N/A indicates that the corresponding functional effect has not been experimentally validated.

Collectively, these findings establish that the ubiquitin system governing ERα stability operates not as a sequential cascade of enzymes with simple, interchangeable redundancy, but as an integrated regulatory network in which apparent redundancy is coupled with mechanistic diversification. Rather than representing mere duplication, this multi-enzyme organization provides regulatory robustness and buffering capacity, while preserving the ability for context-specific fine-tuning. Although E3 ubiquitin ligases are traditionally associated with K48-linked polyubiquitination and proteasomal degradation, the expanding repertoire of ERα-targeting E3 ligases reveals considerable functional diversity beyond unidirectional proteolysis. Stabilizing ligases alone employ at least three distinct biochemical strategies: direct antagonism of K48-linked chains, monoubiquitination, and K63-linked polyubiquitination, frequently engaging distinct receptor domains to confer differential functional outcomes. The TRIM family further exemplifies this diversification, with members such as TRIM24 employing epigenetic recruitment mechanisms independent of ubiquitin transfer. Concurrently, a functionally distinct subset including MDM2 and E6-AP couples proteasome-mediated turnover to transcriptional activation, challenging the conventional view of degradation as merely a termination signal. In parallel, deubiquitinating enzymes that oppose E3 activity exhibit partial functional overlap, yet they are not fully interchangeable. Many DUBs prevent ERα degradation, constituting an apparent redundancy that likely safeguards signaling fidelity. However, individual members additionally enhance transcriptional activity, rescue specific mutants such as Y537S, or remodel chromatin architecture through histone modification. This organized framework, supported by Fig. 3A and Table 1, clarifies how convergent phenotypic outcomes arise from molecular mechanisms that are partially redundant yet mechanistically distinct. By decoding the relationship between ubiquitin chain topology, domain specificity, and functional context, this integrated view provides a foundation for understanding how ERα signaling intensity is calibrated and how endocrine therapy responsiveness may be modulated in breast cancer.

#### Phosphorylation

Phosphorylation of ERα at multiple serine, threonine, and tyrosine residues distributed throughout the receptor integrates diverse extracellular cues (Fig. 3B). While often ligand-dependent, these modifications are also frequently induced by activated kinase signaling pathways, enabling ligand-independent nuclear translocation and target gene transcription (Fig. 1A), thereby playing important roles in breast cancer. Spanning the AF‑1, DNA‑binding, hinge, ligand‑binding, and C‑terminal F domains, these modifications collectively shape receptor conformation, dimerization, DNA binding, co‑regulator interactions, and proteasomal turnover. These context‑dependent modifications either stabilize ERα to enhance transcriptional activity or promote receptor degradation and endocrine resistance^15,183,224^.

The AF-1 domain serves as the principal nexus for ligand-independent activation of ERα, wherein phosphorylation by multiple kinases coordinately enhances receptor stability and transcriptional output. c-Abl-mediated tyrosine phosphorylation at Y52 increases receptor stability^225^, while serine phosphorylation at S104/S106 by ERK1/2 MAPK and at S118 by CDK7, ERK1/2, or GSK-3 collectively enhance both ERα stability and transcriptional activity, thereby contributing to tamoxifen resistance in breast cancer^226–229^. The regulatory architecture surrounding S118 exemplifies the reversible and competitive nature of phosphorylation control: the peptidyl-prolyl isomerase Pin1 recognizes pS118-ERα and stabilizes the protein by disrupting its interaction with the E3 ligase E6AP, thereby inhibiting proteasome-dependent degradation without affecting *ESR1* transcript levels^230^. Conversely, dephosphorylation by PP2A at S118 reduces transcriptional activity^231^. The tumor suppressor p21 normally induces cell cycle arrest, and its loss results in S118 hyperphosphorylation that promotes tamoxifen-stimulated proliferation of breast cancer cells^232^, revealing functional crosstalk between cell cycle regulation and ERα phosphorylation status. A particularly instructive case of context-dependent phosphorylation function is presented by S167. Under estrogen-depleted conditions, PI3K/AKT/mTOR signaling phosphorylates S167, upregulating pS2 and Bcl-2 and suppressing tamoxifen-induced apoptosis^233,234^. Estrogen further amplifies this pro-survival axis by driving S6K1 expression, establishing a positive feedback loop that promotes breast cancer cell proliferation^235,236^. Yet clinical evidence indicates that S167 phosphorylation, strongly associated with p90RSK and MAPK activation, correlates with longer disease-free and overall survival in ERα-positive breast cancer patients and predicts a more favorable response to endocrine therapy^185^. This apparent paradox between in vitro pro-survival signaling and in vivo favorable prognosis suggests that S167 phosphorylation may not function as a unidirectional oncogenic driver; rather, its clinical consequences likely depend on the integrated signaling environment, tumor stage, and compensatory feedback mechanisms that remain incompletely characterized.

Phosphorylation within the DBD directly impinges upon receptor-DNA interaction and reveals a regulatory logic distinct from that of AF-1. c-Abl-mediated phosphorylation at Y219 cooperates with Y52 to enhance receptor stability^225^, indicating that tyrosine phosphorylation in the DBD can parallel the stabilizing function observed in the AF-1 domain. However, PKA-mediated phosphorylation at S236 introduces a more complex regulatory paradigm: this modification inhibits receptor dimerization, which would be expected to attenuate high-affinity DNA binding and transcriptional activity, yet simultaneously protects ERα from ligand-mediated proteasomal degradation^237,238^. This indicates that phosphorylation can reduce transcriptional activity while maintaining protein stability, demonstrating that these functional dimensions are independently regulated. This differs from several AF-1 modifications that concurrently enhance both properties, supporting that phosphorylation outcomes are residue-specific.

The hinge region, historically regarded as a passive linker between the DBD and LBD, functions instead as an active regulatory module in which adjacent phosphorylation sites can enforce diametrically opposed functional states. CK2-mediated phosphorylation at S282 maintains the receptor in a low-activity reserve state by suppressing basal activity. The S282A mutation produces ligand-independent activation, demonstrating that this modification normally restrains constitutive receptor function^239,240^. In primary ERα-positive breast tumors, high pS282 levels are significantly associated with longer relapse-free and overall survival in tamoxifen-treated patients, leading to its classification as a favorable phospho-epitope^239,240^. In direct functional opposition, p38MAPK-mediated phosphorylation at S294 enhances ligand-dependent receptor activity^241^, effectively promoting transition to an active transcriptional state. Calcineurin directly dephosphorylates S294, preventing receptor degradation and enhancing stability, while also indirectly promoting mTORC1-dependent phosphorylation of S118 to augment transcriptional activity^242^. The proximity of S282 and S294 to the DBD further suggests a mechanistic role in modulating DNA binding and/or receptor dimerization. Thus, the hinge region operates not as a passive structural tether but as an integrated signaling hub that determines whether ERα remains in a suppressed reserve configuration or transitions to an active transcriptional state.

The ligand-binding domain (AF-2/LBD) represents a major convergence point for kinase signaling, endocrine resistance, and internally balanced regulatory mechanisms. S305 phosphorylation functions as a critical resistance node targeted by multiple kinases with functionally convergent outcomes: TBK1 phosphorylation enhances transcriptional activity and reduces breast cancer cell responsiveness to tamoxifen^243^; IKKβ-mediated phosphorylation establishes a charge-linked bridge with the F domain that stabilizes helix 12 in a SERM-locked configuration, redirecting signaling toward AF-1-driven constitutive activity and establishing an E2-like cistrome that overrides SERM repression^38,244^; PKA-mediated phosphorylation at the same residue enhances c-MYC expression and upregulates its target genes to promote proliferation in the presence of tamoxifen^245^, while also facilitating co-activator SRC-1 binding to the AF-1 domain to drive hormone-independent proliferation^246^. This multi-kinase convergence upon a single residue underscores S305 as a molecular hub linking diverse upstream oncogenic signals to ERα transcriptional output. Separately, p38MAPK-mediated T311 phosphorylation enhances nuclear translocation and coactivator recruitment independent of ligand-binding status^247^, further expanding the ligand-independent functional repertoire of the LBD. Nevertheless, the AF-2 domain is not exclusively dedicated to activation and stabilization. Cyclin E-CDK2 phosphorylation at S341, specifically activated in the presence of estrogen, enhances ubiquitination and subsequent proteasomal degradation of ERα^248^, constituting an intra-domain counterbalance to the activating modifications at S305 and T311. The Y537 residue further illustrates the ternary interplay between phosphorylation, activating mutations, and dephosphorylation: Src family kinases including c-Src and Lck phosphorylate Y537, a modification required for receptor homodimerization and high-affinity DNA binding^249^. The activating mutations Y537S/N/C, frequently detected in metastatic breast tumors, stabilize an estrogen-independent active conformation by mimicking the electrostatic effects of phosphorylation^64,101^. Conversely, the unphosphorylatable Y537F mutant causes constitutive nuclear retention, abolishes interaction with Ran and Crm1, prevents FKHR nuclear export, reinforces FKHR-mediated repression of cyclin D1, blocks estrogen-induced S-phase entry, and counteracts cell transformation driven by activated Src or PI3K^250^. The reversibility of this modification is demonstrated by the phosphatase PTPH1, which enhances antiestrogen sensitivity by dephosphorylating Y537^251^, highlighting both the dynamic nature of this regulatory node and its potential as a therapeutic target.

The extreme C-terminal F domain, though less extensively studied, contains phosphorylation sites that exert suppressive effects on basal receptor activity. CK2-mediated phosphorylation at S559 suppresses basal ERα activity, and its loss through the S559A mutation results in ligand-independent activation, demonstrating that this modification functions as a negative regulatory constraint. S559 resides near helix 12, and its phosphorylation may influence ERα interactions with coregulators by altering helix-12 positioning, thereby modulating ligand responses. In clinical specimens, pS559 is detectable in a substantial subset of ERα-positive tumors and exhibits hazard ratios above 1 for both overall and relapse-free survival under tamoxifen treatment, leading to its classification as an unfavorable phospho-epitope^239,240^. Notably, both S559 and S282 phosphorylation suppress basal ERα activity, yet their clinical prognostic implications are diametrically opposed, with pS282 predicting favorable and pS559 unfavorable outcomes on tamoxifen. This suggests that the clinical impact of a suppressive phosphorylation event cannot be inferred solely from its effect on transcriptional activity, but likely reflects residue-specific differences in co-regulator interactions, receptor degradation kinetics, or downstream target gene selectivity (Table 1).

The phosphorylation landscape of ERα requires a shift from cataloging individual sites toward understanding integrated regulatory principles. While AF-1 and AF-2 modifications predominantly enhance stability or activity, critical exceptions exist notably S341-mediated degradation in AF-2^248^ and the clinical paradox of S167^185^. Conversely, the hinge region and F domain primarily suppress basal activity, yet adjacent sites such as S282 and S294 exert opposing functions^239–242^. The DBD occupies an intermediate position where stability and activity can be independently regulated, as demonstrated at S236^237,238^. These intra- and inter-domain variations underscore that ERα maintains precise transcriptional control through dynamic equilibrium among reserve, activation, and degradation states, rather than through the dominance of any single modification signal. Endocrine resistance involves dysregulation at multiple sites across these domains rather than resulting from a single modification. Future studies should fully characterize the functional dimensions of partially studied sites, particularly where only stability or activity has been examined, and investigate cross-domain regulatory interactions to guide therapeutic development.

#### Acetylation

Acetylation of ERα at specific lysine residues dynamically modulates receptor stability, DNA binding, and transcriptional activity through the opposing actions of acetyltransferases and deacetylases^252^. Notably, p300-mediated acetylation of ERα occurs at distinct lysine residues with divergent functions. Initially, K302 and K303 within the hinge region were identified as p300 substrates, with these modifications suppressing ligand sensitivity and regulating transcriptional activation^253^. Subsequent quantitative analysis of full-length ERα revealed that K266 and K268 represent the predominant acetylation sites, requiring ligand-bound receptor and SRC-2 coactivator to enhance DNA binding and transactivation^254^. Furthermore, Sung-Hye Kim et al. reported that HDAC inhibitors enhance the acetylation and stability of both ERα and p300, thereby blocking ubiquitination and proteasomal degradation of ERα^255^. SIRT1 deacetylates ERα and represses its transcriptional activity, whereas DBC1 (CCAR2) and CCAR1 cooperatively inhibit SIRT1, thereby maintaining ERα acetylation and enhancing its DNA binding and transcriptional activity^256,257^. It is noteworthy that phosphorylation of ERα at the S305 residue suppresses acetylation at the K303 site and elicits an enhanced transcriptional response^184^. In addition, BRCA1 depletion has been shown to increase ERα acetylation in a PI3K/Akt‑dependent manner, at least partly through up‑regulation of the coactivator p300^258–260^. These acetylation events engage in functional crosstalk with phosphorylation and ubiquitination pathways to precisely govern receptor turnover and transcriptional activity. Targeting the enzymatic machinery that mediates ERα acetylation thus offers potential strategies to restore endocrine sensitivity in resistant breast cancer (Fig. 3C).

#### Methylation

Methylation of ERα at lysine and arginine residues establishes a site-specific regulatory code that parallels histone modification logic, wherein methyltransferases and demethylases antagonistically mark the receptor with distinct functional indices governing stability and transcriptional activity. Lysine methylation functions as a bidirectional switch. In the activating mode, SET7-mediated methylation at K302 enhances ERα protein stability and promotes its recruitment to target gene promoters, augmenting transcriptional output^261^. Likewise, G9a-catalyzed dimethylation at K235 generates a recognition signal for the PHF20-MOF acetyltransferase complex, which subsequently deposits H4K16 acetylation at target promoters to amplify gene expression^262^. In contrast, K266 methylation enforces transcriptional silencing in the resting state; upon estrogen stimulation, this mark is actively removed, thereby permitting CBP/p300-mediated acetylation at the same residue and switching ERα to an active conformation. Accordingly, SMYD2 overexpression elevates K266 methylation to suppress ERα acetylation and chromatin occupancy, whereas demethylase LSD1 reverses this repressive state^263^. Moreover, the functional output of these methylation marks is further refined by competitive interplay with spatially adjacent phosphorylation events. The functional consequences of lysine methylation are further refined by competitive interplay with spatially adjacent phosphorylation, wherein the immediate adjacency of K235 and S236 allows these marks to antagonistically regulate ERα dimerization and DNA recruitment, processes that are disrupted by S236 phosphorylation to ultimately determine net transactivation activity^264^. More broadly, KDM2A removes repressive lysine methylation from ERα, and this erasure is required for the receptor to undergo activating phosphorylation at S118 and S167^265^. This sequential demethylation-to-phosphorylation cascade shifts the receptor toward an active transcriptional state. Arginine methylation couples cytoplasmic signaling to chromatin architecture. Beyond lysine residues, PRMT1 catalyzes R260 methylation in the cytoplasmic pool of ERα upon E2 stimulation, creating a docking platform for the PI3K/Src/FAK scaffolding complex that activates Akt signaling to drive breast cancer progression^266^. Intriguingly, the putative arginine demethylase JMJD6 adds regulatory complexity by performing a dual function: although capable of demethylating R260, JMJD6 is simultaneously required within the CARM1/MED12/ERα complex to establish enhancer-promoter looping, thereby linking arginine methylation dynamics to three-dimensional chromatin topology and target gene control^267,268^ (Fig. 3D). Together, these findings illustrate that ERα methylation operates not as a static modification but as a dynamic, residue-specific regulatory language. The balance between methyltransferase and demethylase activity, modulated by extensive cross-talk with phosphorylation and acetylation, determines receptor stability, subcellular partitioning, and transcriptional competence in a context-dependent manner.

#### SUMOylation

SUMOylation of ERα operates as a ubiquitin-like modification that modulates receptor DNA binding and transcriptional output in a ligand-dependent manner, with distinct consequences for endocrine drug responses^269^. Two ligand-dependent SUMOylation sites, K266 and K268, have been identified in the ERα hinge region, enhancing ERα target gene expression in vitro. PIAS1 and PIAS3 act as E3 ligases for ERα SUMOylation in the presence of estrogen, maintaining ERα’s DNA-binding ability and promoting transcription^270^. TRIM3-induced SUMOylation of ERα through its interaction with ubiquitin carrier protein 9 (UBC9) enhances the transcription of ERα-target genes, contributing to tamoxifen resistance in BCa^271^. Fulvestrant induces SUMOylation of ERα, mainly at K171 and K180 (with additional modification at K99 and K472), causing transient DNA binding followed by rapid release and complete transcriptional shut‑off. SENP1 counteracts this by delaying receptor dissociation and partially restoring target gene expression. By contrast, tamoxifen does not trigger ERα SUMOylation, and the receptor remains bound to chromatin with altered cofactor recruitment, leading only to partial repression^109,272^ (Fig. 3E).

#### Other post-translational modification of ERα

Additional PTMs broaden ERα regulation beyond the canonical marks. The palmitoylation of cysteine 447 (C447) in ERα is mediated by DHHC-7 and DHHC-21, promoting its interaction with caveolin-1 and subsequent translocation to the plasma membrane, thereby initiating nongenomic ERK/MAPK signaling^273–275^. O‑GlcNAcylation of ERα at residues T553 and S554 by GREB1 prevents the recruitment of the E3 ubiquitin ligase ZNF598, thereby suppressing ERα ubiquitination and subsequent degradation^276^. GALNT6 mediates O‑GalNAc glycosylation at the F‑domain residue S573 of ERα, and this modification stabilizes the receptor and promotes its nuclear localization, thereby reinforcing ERα signaling in breast cancer cells^277^. In normal mammary epithelial cells, ERα undergoes O‑GalNAc glycosylation at Serine 10 (S10). In TNBC cells, however, aberrant UGT1A1 expression stalls this site at the initial UDP‑GlcNAc glycosylation step, preventing completion of the normal glycosylation maturation. This incomplete modification reduces ERα protein abundance and impairs estrogen responsiveness^278^ (Fig. 3F).

ERα undergoes diverse post-translational modifications that converge on common regulatory nodes, including protein stability, DNA binding, and cofactor recruitment, to shape transcriptional output. These marks form an interconnected network where modifications at specific residues, notably K266 and K302/303, exhibit extensive crosstalk, competing or cooperating to determine receptor fate. Across distinct modifier families, a recurring organizational principle emerges: enzymes producing similar functional outcomes, whether stabilization, degradation, or transcriptional potentiation, rarely represent redundant duplicates. Instead, they typically employ distinct molecular mechanisms, as exemplified by E3 ligases utilizing varied ubiquitin chain topologies, DUBs acting through deubiquitination, co-activation, or chromatin modification, and KDMs licensing receptor activity via phosphorylation priming or acetylation facilitation. This mechanistic diversification within functional convergence implies that inhibiting a single enzyme may be circumvented by compensatory activity from mechanistically distinct relatives, limiting single-agent efficacy. While HDAC and kinase inhibitors have advanced to clinical application, targeting specific enzymes governing methylation, ubiquitination, or emerging PTMs remains largely preclinical. Consequently, therapeutic strategies that simultaneously address multiple nodes within this network may be required to resensitize tumors to endocrine therapy and overcome acquired resistance.

### Estrogen receptor β in BCa

In ERα-positive breast tumors, ERβ, especially the full-length ERβ1 isoform, is frequently co-expressed with ERα, yet large cohort analyses reveal a trend toward inverse correlation at both mRNA and protein levels^279,280^. Population-based studies demonstrate that high ERβ1 immunoreactivity is an independent predictor of favorable clinicopathological features: lower histological grade, low Ki-67 index, negative nodal status and prolonged disease-specific survival among patients treated with adjuvant chemotherapy^280^. Importantly, the prognostic value appears restricted to ERβ1; alternative splice variants such as ERβ2 (ERβcx) lack the ligand-binding helix 12 and may even lose tumor-suppressive capacity^281,282^. Although pre-clinical data suggest that ERβ re-expression sensitises tamoxifen-resistant cells to anti-estrogens^283^, the only population evidence so far links ERβ1 abundance to chemosensitivity rather than to endocrine response. Thus, current clinical biomarker development focuses on ERβ1 as a chemotherapy-related prognosticator, while its predictive role for endocrine therapy awaits prospective validation.

ERβ curtails ERα signaling at multiple regulatory layers. By preferentially forming transcriptionally weak ERα/ERβ heterodimers, ERβ reduces the pool of active ERα homodimers available to canonical oestrogen-response elements, leading to diminished expression of proliferation genes such as CCND1 and MYC^284^. Genome-wide studies further show that ERβ recruits co-repressor complexes containing HDACs and NCoR to ERα-occupied enhancers, converting them into facultative heterochromatin^283,285^. Parallel nongenomic actions include transcriptional down-regulation of HER2/HER3 and concomitant induction of PTEN, which collectively blunt PI3K-AKT and MAPK-ERK cascades that normally potentiate ERα activity^286^. More recently, ERβ was shown to engage AGO2 and the spliceosome, promoting alternative splicing and R-loop formation at ERα target loci; the resulting transcription-replication conflicts activate a DNA-damage program that can trigger apoptosis, particularly in highly proliferative tumors^285,287^. Hence, ERβ functions both as a passive competitor and as an active reprogrammer of ERα chromatin signaling.

ERβ protein is subjected to tightly coordinated post-translational modifications that calibrate its half-life and transcriptional output. MDM2 acts as the major E3 ubiquitin ligase, poly-ubiquitylating ERβ and targeting it for 26S proteasomal degradation, whereas the acetyl-transferases CBP/p300 antagonize this process by acetylating multiple lysine residues within the hinge region^288^. Phosphorylation of Tyr-36 by Src-family kinases operates as a functional switch: unphosphorylated ERβ behaves as a tumor suppressor, whereas Tyr-36 phosphorylation converts the receptor into a pro-survival factor and overrides its growth-inhibitory genome-wide program^289^. At the epigenetic level, methylation of the ESR2 promoter and repressive histone marks (H3K27me3) silence ERβ in a subset of ERα-positive tumors; this silencing can be reversed clinically by DNA-methyl-transferase or HDAC inhibitors, restoring tamoxifen sensitivity in xenograft models^283^. Collectively, these layers create a dynamic rheostat that determines whether ERβ will reinforce or resist estrogen-driven proliferation.

Pre-clinical studies provide a strong rationale for ERβ-directed therapy. Selective ERβ agonists such as DPN, 8β-VE2 or LY500307 cooperate with tamoxifen or fulvestrant to inhibit colony formation, trigger caspase-3-mediated apoptosis and delay acquired resistance in long-term oestrogen-deprivation models^283,286^. Combination with chromatin-modifying agents (e.g., entinostat, vorinostat) further enhances ERβ re-expression and re-sensitizes resistant tumors to endocrine therapy. Conversely, analysis of TCGA data and isogenic mouse models indicates that TP53 mutation abrogates the tumor-suppressive program of ERβ and may even divert the receptor toward pro-proliferative transcription; in this setting, ERβ antagonism rather than activation could be beneficial^290^. Finally, emerging evidence that ERβ activation augments CD8⁺ T-cell receptor signaling and dampens metastatic dissemination of inflammatory BCa suggests that ERβ agonists might synergise with immune-checkpoint blockade^291,292^. Ongoing phase-I/II trials are therefore evaluating oral ERβ agonists as chemo-endocrine sensitizers and immuno-modulators, with integrated TP53 sequencing to guide patient stratification.

Collectively, ERβ operates as a transcriptional gatekeeper that restrains ERα-mediated transcription. Its maintained expression signifies a less aggressive ERα-positive phenotype and constitutes a mechanistically grounded co-target for restoring endocrine sensitivity or enhancing immuno-chemotherapeutic efficacy.

Estrogen receptor signaling in breast cancer operates through a multi-layered regulatory architecture where ERα serves as a convergent node integrating genomic alterations, epigenetic marks, and post-translational modifications to determine transcriptional output. While ERβ-mediated antagonism represents an additional regulatory constraint, its mechanistic basis and clinical utility remain to be fully elucidated. Current understanding is limited regarding the hierarchical coordination of competing modifications at hotspot residues, the context-dependent interplay among mechanistically distinct modifying enzymes, and the dynamic transitions governing the evolution from reversible adaptation to irreversible resistance. Future investigations would benefit from moving beyond single-modifier targeting to explore context-dependent synthetic lethal interactions and to develop multi-omic biomarker frameworks that capture the combinatorial complexity of the ERα regulatory network. Such mechanism-guided approaches may inform the design of rational combination therapies potentially capable of disrupting adaptive reprogramming and restoring sensitivity to endocrine intervention in resistant tumors.

## Progesterone receptor signaling pathway in BCa

As noted above, the PGR gene gives rise to two major progesterone receptor isoforms, PRA and PRB, with PRB representing the full-length receptor and PRA produced from an alternative upstream promoter, lacking 164 amino acids at the N-terminus. In normal breast, PRA and PRB are expressed at equimolar ratios, yet this balance is altered in BCa, where an increased PRA/PRB proportion has been reported to correlate with aggressive disease and diminished disease‑free survival^4,29,293^. PRB mediates the predominant progesterone‑induced transcriptional activity, whereas PRA alone shows minimal activity but can attenuate PRB-dependent progesterone responsiveness when co‑expressed^31,294^. This inhibitory effect probably reflects an amino-terminal inhibitory domain active in PRA that is masked in PRB, thereby enabling PRA to suppress not only PRB but also ER activity^295^. Consistently, individual analysis reveals markedly distinct transcriptional signatures for PRA and PRB in breast cancer cells, indicating complementary physiological functions. Upon co-expression, they form heterodimers and homodimers that coordinately regulate distinct and shared targets^295^. Under progestin-independent conditions, PRA is more active than PRB and is predominantly localized in the nucleus, yet it fails to mediate direct transcriptional events or rapid cytoplasmic changes^10,296^. Moreover, variation in the PRA/PRB ratio carries distinct biological implications: higher ratios align with luminal A features and anti-progestin sensitivity, whereas lower ratios correspond to luminal B characteristics, increased proliferative signaling, and reduced or even stimulatory responses to anti-progestin treatment^297^.

Progesterone receptor signaling exhibits marked functional plasticity in ERα-positive breast cancer, capable of either promoting malignant progression or enforcing growth restraint depending on cellular context. This context dependence reflects the combinatorial influence of PRA/PRB stoichiometry, ligand identity, epigenetic co-regulator assembly, and post-translational modifications. Central to this regulatory complexity is the extensive crosstalk between PR and ERα, which enables PR to function not merely as a parallel effector but as a dominant determinant of ERα chromatin binding and transcriptional output, either reinforcing or antagonizing estrogen-driven proliferation^4,298–301^. The following sections examine these interconnected mechanisms, beginning with the dual pathological roles of PR in breast cancer, followed by chromatin-level co-regulation and post-translational control.

### Dual face of PR in ERα-positive BCa

#### The carcinogenic role of PR

The carcinogenic role of PR has been supported by several lines of evidence. PR was first implicated in breast carcinogenesis through its ability to sustain cyclical expansion of mammary stem-cell pools and latent tumor-initiating cells within the mature epithelium^302^. In BRCA1-mutant models, PR protein is up-regulated and the antagonist mifepristone blocks proliferation; long-term administration prevents mammary tumor formation^303^. Progesterone-PR signaling can also downregulate major histocompatibility complex (MHC) class I expression, thereby promoting immune evasion and enabling escape from immune-mediated clearance of ERα+PR+ BCa^304^. These findings provided the rationale for translating anti-progestins into the clinical testing^4^.

A critical modifier of PR signaling in breast cancer is the relative abundance of PRA and PRB. In ERα+BCa patients, tumors with a high proportion of PRA (PRA-rich) are significantly associated with a shorter disease-free survival period^305^. Classic studies demonstrated that transcriptionally inactive PRA can act as a potent hormone-dependent trans-dominant repressor of PRB mediated transcription. This inhibition persists even when PRA is present at substoichiometric levels relative to PRB and does not require DNA binding. PRA can also suppress the transcriptional activity of GR, AR, and MR, but not that of the non-steroid VDR, indicating a selective regulatory function. A plausible mechanism is that PRA binding restricts access to transcriptional cofactors required for PRB activity^306^. PRB is associated with differentiation‑linked and growth‑restraining transcriptional programs, and excess PRA can suppress these PRB mediated outputs, a shift that is linked to reduced endocrine responsiveness and tamoxifen resistance^305^.

The functional consequences of PRA dominance are further illustrated in ligand-dependent transcriptional studies. In PRA-only T47D model cells, the synthetic progestin medroxyprogesterone acetate recruits AIB1 to CCND1 and MYC promoters to accelerate G1-S transition, whereas mifepristone recruits the corepressor SMRT to the same loci, silencing transcription and inhibiting growth. However, mifepristone does not inhibit proliferation in PRB-only T47D cells^307^. Consistent with these findings, the phase II MIPRA trial demonstrated that mifepristone induces G1 arrest, apoptosis, and reversal of immune exclusion specifically in ERα+ breast cancers with a high PRA to PRB ratio^308^.

Additional studies highlight distinct biological behaviors of PRB dominant tumors. In PRB-only models, PRB upregulates VEGF even in the presence of tamoxifen or fulvestrant, whereas PRA suppresses this effect, suggesting that PRB dominant ERα+ breast cancers may benefit from antiangiogenic therapy due to better developed vasculatures^309^. Despite this, both progestin and mifepristone can enable PRB to drive cell cycle progression in ERα+ breast cancer cells through activation of cytoplasmic MAPK signaling, independently of direct transcriptional regulation^310^. Another study shows that in ERα+ breast cancer cells expressing only PRB, mifepristone binding triggers PRB phosphorylation, which promotes sustained PRB occupancy at stemness gene enhancers such as SOX2 and NANOG, thereby maintaining their expression and driving cancer progression^311^.

Paradoxically, these findings limit the therapeutic potential of mifepristone. Nevertheless, they establish the PRA/PRB ratio as a predictive biomarker, shifting the binary “oncogenic versus tumor-suppressive” paradigm toward a precision medicine-guided framework for identifying patients most likely to benefit from PR antagonism.

#### The tumor-suppressive role of PR

Recent work shows that PR expression can serve as a positive prognostic indicator. PR negativity in ERα+BCa identifies a high-recurrence subset, and ER⁺PR⁺ cancers respond better to SERMs than ERα+PR- lesions^312^.

The effects of different progestogens on the risk of BCa vary. Progestins like MPA are reported associated with an increased risk of BCa. Progesterone (P4), however, does not increase the risk and may even reduce it^313^. Megestrol acetate, a synthetic progestin, shows clinical activity in ERα+ metastatic patients who have failed aromatase-inhibitor therapy^314^. Progestin (R5020) induce an immediate proliferative burst in ERα+PR+ cells within a single cell cycle, but this is followed by sustained growth arrest^301^. Mechanistically, progestin-activated PR relies on pre-bound C/EBPα, which maintains open chromatin and facilitates PR recruitment to enhancer regions, with partial contribution from FOXA1 at selected sites. In parallel, chromatin architectural proteins such as CTCF and Cohesin stabilize enhancer–promoter loops, ensuring the activation of a regulatory network that includes antiproliferative genes such as DUSP1, thereby mediating progesterone-induced growth suppression in breast cancer cells^301^. In ERα+ patient-derived xenografts, PR was found to co-localize with RNA Polymerase III at tRNA gene promoters. Progestogen treatment reduced the abundance of select pre‑tRNAs and mature tRNAs, indicating that PR can modulate Pol III-dependent transcription. By limiting the availability of tRNAs required for protein synthesis, PR thereby contributes to the suppression of breast cancer growth^315^.

#### The crosstalk between PR and ERα

**Fig. 4:**
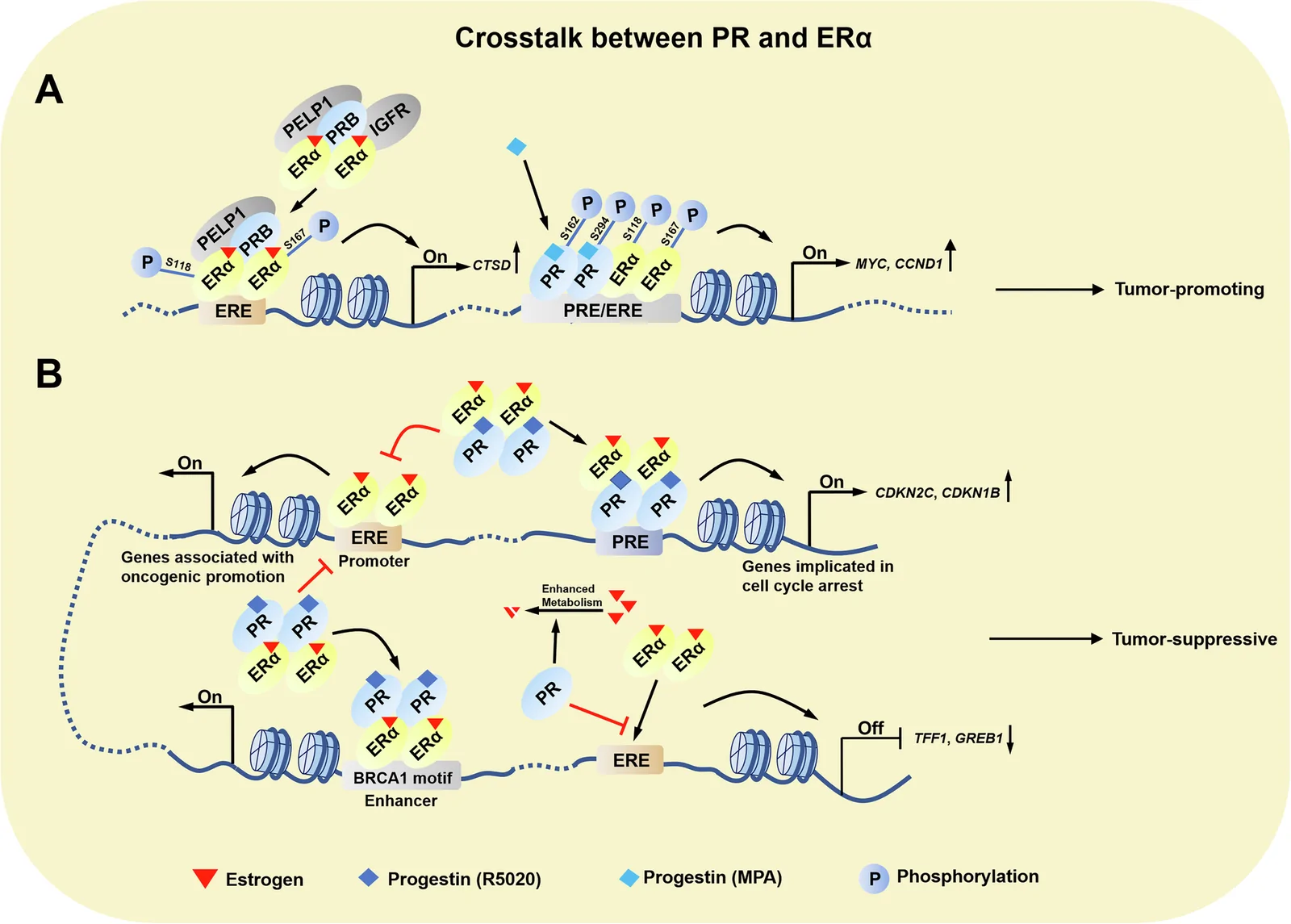
Crosstalk between PR and ERα signaling pathways in breast cancer. **A** PR‑B scaffolds ERα, PELP1, and IGF1R to enhance ERα phosphorylation and estrogen‑driven proliferation. MPA promotes phosphorylation of PR (S162/S294) and ERα (S118/S167), facilitating nuclear PR-ERα complex formation, thereby driving oncogenic transcription. **B** In the presence of both estrogen and progestin, PR reprograms ERα chromatin binding toward PRE-enriched loci associated with cell-cycle arrest, and redirects ERα to distal enhancers enriched for BRCA1 motifs, exerting tumor-suppressive effects. PR overexpression further impairs ERα binding to EREs and accelerates estrogen uptake and metabolism, thereby attenuating ERα signaling and suppressing proliferation. ERE estrogen response element, PRE progesterone response element, BRCA1 motif BRCA1-enriched DNA binding sequences, P phosphorylation, MPA Medroxyprogesterone acetate (synthetic progestin).

During mammary gland development, estrogen-ERα and progesterone-PR signaling operate in a stage dependent manner. Estradiol-driven ERα activity is essential for early ductal elongation, whereas progesterone‑dependent PR signaling becomes required for continued ductal growth and side‑branching as estrogen levels rise. PR and ERα share overlapping transcriptional programs and exhibit extensive crosstalk, with PR capable of both reinforcing and restraining ERα-driven oncogenic activity depending on cellular context and hormonal environment. This bidirectional interplay positions PR as a critical modulator of estrogen action in breast cancer^10,299,316,317^. In addition, the docking study reporting higher predicted affinity of estradiol and estrone for PR provides a conceptual indication that, in an estrogen‑rich environment, additional layers of ERα-PR crosstalk may occur beyond simple ligand-receptor specificity^318^ (Fig. 4).

PRB overexpression in ERα+ breast cancer cells enhance estrogen responsiveness independently of progestin. PRB functions as a scaffold that assembles ERα, PELP1, and IGF1R into a signaling complex. This arrangement enhances IGF1R/PI3K-mediated phosphorylation of ERα (S118/S167), thereby amplifying estrogen-driven transcriptional programs and proliferation. Targeting PRB partially restores tamoxifen sensitivity in resistant cells^319^. In progestin‑dependent breast cancer, treatment with MPA promotes phosphorylation of PR (S162/S294) and ERα (S118/S167), leading to the formation of nuclear PR-ERα complexes. These activated complexes co‑occupy the *CCND1* and *MYC* promoters, thereby enhancing transcriptional activity and driving tumor cell proliferation^320^.

PR signaling exerts context-dependent effects on ERα activity in breast cancer. In ERα+PR+ tumors, activation of PR by natural or synthetic ligands can antagonize estrogen-driven proliferation. Mechanistically, ligand-bound PR physically associates with ERα and reprograms its chromatin binding profile, redirecting ERα away from canonical proliferation-associated loci toward PRE-enriched regions linked to differentiation, apoptosis, and cell-cycle arrest. This redistribution occurs largely independent of classical EREs and is accompanied by recruitment of co-regulators such as p300, resulting in a transcriptional program associated with favorable clinical outcome^300^. By contrast, enforced or ligand-independent PR expression in ERα+ breast cancer cells elicit a potent antiestrogenic effect. In this setting, PR impairs ERα-mediated transcription by disrupting ERα occupancy at EREs, attenuating induction of canonical estrogen target genes, and accelerating estradiol uptake and metabolism. These combined mechanisms abolish estrogen-induced cell cycle progression and proliferation, thereby establishing a PR-driven, estrogen-depleting pathway that suppresses tumor growth independently of progestin activation, a process predominantly mediated by PRA^10,321^. In ERα+ breast cells, the synthetic progestin R5020-activated PR alters chromatin accessibility and directly interacts with ERα, redirecting it from proximal promoter regions to distal enhancers enriched for BRCA1 motifs. Although PR and ERα act as genomic co‑agonists, this reprogramming shifts ERα activity away from estrogen-driven proliferative programs toward transcriptional outputs linked to differentiation and apoptosis, thereby exerting phenotypic antagonism and positioning PR as the dominant determinant of ERα functional output. Functionally, such PR-mediated remodeling underlies the marked regression of ERα+PR+ xenografts when the selective PR modulator/antagonist Telapriston is combined with tamoxifen, supporting combined ERα/PR targeting as a therapeutic strategy^299^ (Fig. 4).

Beyond direct transcriptional interference, PR also modulates estrogen‑dependent tumor growth through ERα-independent mechanisms. In ERα+ breast tumors, PR not only directly attenuates ERα-driven transcription but also engages RNA Polymerase III at tRNA gene loci, where progestogen treatment reduces tRNA abundance and thereby constrains translational capacity, collectively contributing to the suppression of estrogen-dependent tumor growth^315^. In antiprogestin-responsive (PRA-rich) tumors, carcinoma associated fibroblast derived FGF2 activates FGFR signaling to phosphorylate PR and ERα in a ligand-independent manner. This may promote the formation of transcriptional complexes comprising STAT5, ERα, PR, and PRBΔ4 (PR variants) at PRE, sustaining target gene transcription and potentially contributing to endocrine resistance^322^. PRMT1 methylates ERα at Arg260 and PR at Arg637, positioning this enzyme as a shared upstream regulator of both receptors. Its dual actions on ERα‑dependent rapid signaling and PR-dependent transcriptional turnover suggest a mechanistic link that may contribute to ERα-PR crosstalk^10^.

Collectively, these findings indicate that ERα-PR crosstalk is not fixed but highly dynamic, oscillating between synergistic and antagonistic states. The functional outcome of this interplay is dictated by multiple variables, including the relative abundance of hormones, the chromatin landscape, and the cellular milieu. Such context-dependent modulation underscores PR’s role as a pivotal determinant of ERα signaling output in breast cancer.

### Epigenetic co-regulation of PR-induced transcription in BCa

Analogous to ERα signaling, PR-mediated transcription is orchestrated by an intricate cohort of epigenetic coregulators^10^. In the absence of ligand, unliganded PR collaborates with the long non-coding RNA SRA to nucleate a repressive complex composed of HP1γ, LSD1, HDAC1/2, CoREST, and KDM5B at PREs of hormone-inducible genes, thereby locking chromatin in a transcriptionally silent state^323^. Ligand binding triggers rapid recruitment of ERK and MSK1 kinases by activated PR, catalyzing histone H3S10 phosphorylation that evicts the repressive complex and licenses transcription^324^. PR then exchanges KDM5B for the NURF remodeler or the ASCOM methyltransferase complex, leading to elevated H3K4me3 and enabling phosphorylation by Cdk2-cyclin A, which displaces linker histone H1 and thereby opens chromatin^325,326^. Concurrently, PCAF or BAF complexes deposit H3K14ac and execute ATP-dependent eviction of H2A/H2B dimers, events indispensable for full activation of progesterone target genes^325,327^. High-occupancy nucleosomes at ERα-enriched PR binding sites are remodeled within minutes, leading to systematic removal of H1 and H2A/H2B^325,327^. Progesterone also represses a distinct subset of genes. Ligand-activated PR retains HP1γ-LSD1 but supplements it with the methyltransferase SUV39H2, which installs H3K9me3 to establish facultative heterochromatin^323,328^. This repressive program is further reinforced by selective recruitment of the ATPase BRG1 (independent of the remainder of the BAF complex), which partners with histone H1.2 to compact chromatin and impede RNA polymerase II loading; FOXA1 pre-marks these promoters for hormonal silencing^328^. Accordingly, activated genes exhibit MLL2/3-dependent H3K4me3 enrichment and prominent H3K14ac^323,326^, whereas repressed loci accumulate SUV39H2-catalyzed H3K9me3 and undergo HDAC1/2-mediated deacetylation of H3K27 and H3K16 without appreciable changes in H3K14ac^323,328^. Architectural analysis reveals that activating PREs reside in distal enhancers, while repressive PREs cluster proximally to transcription start sites, underscoring promoter-enhancer specificity in PR-directed chromatin remodeling^327,328^.

At the immune‑modulatory B7‑H4 enhancer, progesterone recruits p300‑BRD4, increases H3K27ac and boosts B7‑H4 transcription, leading to elevated B7‑H4 expression and reduced T‑cell activity in vitro. By contrast, PR does not occupy PD‑L1 regulatory regions^329^. The glucocorticoid receptor competes with PR for shared genomic loci and antagonizes progesterone-dependent expression of proliferation and EMT genes^330^. BRCA1, an E3 ubiquitin ligase that interacts directly with PR, is recruited to PREs, increases monoubiquitinated H2A and contributes to epigenetic silencing of PR target oncogenes^331^.

### Post-translational modification of PR in BCa

Phosphorylation and SUMOylation are the best-characterized PR modifications. Phosphorylated PR displays transcriptional activation, whereas SUMOylated PR recruit co-repressors that curb its activity^332^. S294-phosphorylated PR is abundant in ERα+PR+ BCa and drives transcription of *SLC37A2*, *RUNX2*, *HER2*, *PAX2* and *AHR*, up-regulating a lobular-like gene signature and increasing mammosphere formation frequency^311^. MAPK and CDK2 phosphorylate PRB, but not PRA, at Ser294^332,333^. This phosphorylation antagonizes ligand-dependent SUMOylation at Lys388, so that deSUMOylated receptors are preferentially recruited to enhancers of SUMO-sensitive genes (e.g., MSX2, RGS2, MAP1A, PDK4), where they cooperate with CBP and MLL2 to promote proliferation and survival^334^. Additional CDK2-target residues (Ser190, 345, 400, 554, 676) mediate ligand-dependent and -independent gene transcription^332,335,336^. Phosphorylation of S81 by CK2 is essential for progesterone-independent proliferation and for induction of anti-apoptotic BIRC3^337^. Once phosphorylated, PR is ubiquitinated and rapidly degraded by the 26S proteasome^338^. The BRCA1/BARD1 complex acts as an E3 ubiquitin ligase that inhibits PR transcriptional activity through dual mechanisms: it directly promotes PR ubiquitination to reduce PR protein stability and promoter recruitment, and upon hormone stimulation, it is recruited to PR target gene promoters where it mediates histone H2A monoubiquitination and epigenetic silencing, thereby providing a mechanistic basis for the tissue-specific breast cancer predisposition associated with BRCA1 mutations^331^. Acetylation of K183 by SRC-1/p300 enhances PR-dependent transcription initiation^339^, while PRMT1 methylation at R637 increases receptor recycling and transcriptional output, boosting proliferation and migration^340^. Mono-methylation of K464 in the AF1 domain of PRB diminishes interaction with NCoR1 and SRC-1 and reduces both ligand-dependent and -independent activity^341^.

In summary, PR signaling promotes normal mammary epithelial expansion during development, yet this physiological function is co-opted in breast cancer to drive malignant progression. The PRA/PRB isoform ratio represents one of the principal determinants governing PR functional output, with altered stoichiometry significantly influencing disease behavior and therapeutic responsiveness. Rather than serving as a passive downstream effector, PR functions as a critical contextual modulator of ERα signaling, redirecting estrogen-driven transcriptional programs toward proliferation or growth restraint depending on hormonal context. Epigenetic coregulator dynamics and post-translational modifications provide additional regulatory tuning within this framework. This functional plasticity implies that both progestins and anti-progestins possess context-specific therapeutic value, necessitating individualized treatment strategies. Given that PR signaling is functionally intertwined with the ER-estrogen axis, combined targeting of PR alongside endocrine therapy offers a rational direction for improving patient outcomes. Nevertheless, the development of refined biomarkers beyond PRA/PRB ratios and a comprehensive understanding of the contextual cues that toggle PR between oncogenic and tumor-suppressive states remain critical unmet needs.

## Androgen receptor signaling pathway in BCa

In breast cancer, androgen receptor (AR) expression shows distinct prevalence and prognostic implications across subtypes. AR is detected in ~60–90% of ERα+ tumors, where it generally acts as a tumor suppressor and is associated with favorable outcomes. In contrast, about 70% of HER2+/ERα- tumors express AR, but here AR functions as an oncogenic driver, correlating with poorer disease-free and overall survival. AR is detected in ~10–50% of triple‑negative breast cancers (TNBC), and AR positivity has been associated with poorer clinical outcomes. TNBC represents a highly heterogeneous subtype rather than a uniform ER-negative entity and can be further divided into four transcriptomic subgroups—luminal androgen receptor (LAR), basal‑like 1 (BL1), basal‑like 2 (BL2), and immunomodulatory (IM). Among these, the LAR subtype accounts for about 10% of TNBC and shows nearly a tenfold higher AR expression than other subtypes, together with apocrine-like differentiation, lower proliferative activity, and a characteristic pattern of axillary lymph‑node involvement. Notably, reported AR-positive rates vary across studies, partly due to differences in immunohistochemical scoring criteria and positivity cutoffs^34,342,343^. Alternative splicing and ligand-independent activation generate constitutively active AR variants (AR-Vs, notably AR-V7) that lack the LBD and remain refractory to androgen-deprivation therapies. These variants were first identified in prostate cancer and are also enriched in TNBC, where they correlate with stem-like phenotypes and chemotherapy resistance^5,344^. Despite these seemingly disparate subtype-specific manifestations, AR function remains fundamentally context-dependent^345^.

Although AR and its variants represent established oncogenic drivers in prostate cancer, their roles in breast cancer are considerably more complex and fundamentally context-dependent. In both ERα-positive and ERα-negative subtypes, AR exhibits paradoxical, dualistic functions, acting as either a tumor suppressor or a carcinogenic driver depending on cellular context, hormonal environment, and crosstalk with other signaling axes^7,346–349^. The multifaceted mechanisms underlying these context-dependent roles are examined below.

### The paradoxical role of AR in ERα-positive BCa

#### The tumor-suppressive role of AR

Higher AR levels are associated with improved overall survival (OS) and disease-free survival (DFS), suggesting that AR may serve as a predictive or prognostic biomarker and a potential therapeutic target in ERα-positive BCa^350,351^. Elevated levels of AR are also associated with a more favorable response to tamoxifen in both primary and metastatic ERα-positive breast carcinomas^352–354^. A phase II trial (NCT02463032) demonstrated that AR agonist Enobosarm exhibits anti-tumor activity in patients with ERα-positive advanced BCa, indicating that AR activation may confer clinical benefit^355^. DHT-activated AR engages the p300/SRC-3 transcriptional coactivator complex at AREs, promoting the transcription of AR target genes with putative tumor-suppressive functions^347^. In parallel, AR also binds ARE and Sp1-binding region of the cyclin D1 promoter, displacing RNA Polymerase II and facilitating the recruitment of a corepressor complex comprising DAX1 and HDAC1, which transcriptionally silences cyclin D1, thereby inhibiting ERα + BCa cells proliferation^356^. In addition, mibolerone, a synthetic non-metabolizable androgen, activates AR to enhance ERβ transcription, thereby suppressing ERα+ breast cancer cell growth via p21 upregulation and cyclin D1 downregulation^357^. As ERβ overexpression also downregulates HER2/HER3 and upregulates PTEN to reduce Akt signaling^286^, AR-mediated ERβ induction may potentially suppress tumor growth through these additional downstream mechanisms.

#### The carcinogenic role of AR

While several studies have indicated that the AR signaling pathway functions as a driver of BCa. Androgen activates AR signaling pathway, promoting the proliferation of MCF7, while the AR antagonist enzalutamide effectively inhibits this effect^358^. DHT has been shown to enhance EMT in ERα + BCa by epigenetically regulating the expression levels of E-cadherin and vimentin^346^. The overexpression of AR contributes to ERα + BCa bone metastasis and disrupts the efficacy of tamoxifen and aromatase inhibitor therapies^359–361^. DHT activates AR binding to a distinct cistrome enriched for FOXA1 motifs, promoting transcription of canonical AR target genes such as *FKBP5*. Enzalutamide also enhances the efficacy of tamoxifen and fulvestrant, and in vivo, suppresses tumor growth and metastasis, including in tamoxifen-resistant models^362^.

#### Switch of AR-ERα crosstalk and AR ligand functions in breast cancer

The interplay between AR and ERα in breast cancer is highly dynamic and context-dependent. AR can either cooperate with or reprogram ERα activity, with the functional outcome influenced by the ligand availability, stimulation duration, and the AR/ERα ratio.

Under estradiol stimulation, AR is redirected to ERE-enriched loci, where its presence enhances ERα binding and transcriptional activity, thereby amplifying proliferative signaling in ERα+AR+ breast cancer cells. Therapeutically, enzalutamide disrupts this cooperative interaction by blocking AR nuclear localization, attenuating ERα genomic occupancy and synergizing with tamoxifen and fulvestrant to suppress tumor growth. In vivo, enzalutamide reduces tumor burden and metastasis, including in tamoxifen-resistant models^362^. Beyond driving proliferation, AR-ERα cooperation also extends to metabolic regulation, wherein AR, ERα, and FOXA1 assemble on composite promoter element consisting of a 5′ half-ERE (bound by ERα), a central FOXA1-binding site (FXRE), and a 3′ half-ERE (bound by AR), thereby upregulating steroid-metabolizing enzymes including UGT2B15 and UGT2B17. This transcriptional program correlates with improved survival outcomes in breast cancer^363^. Additional mechanistic studies have demonstrated that activation of the androgen-AR signaling axis can antagonize ERα-mediated transcription and suppress proliferation in ERα-positive breast cancer cells^347,364^. Conversely, androgen-activated AR can antagonize ERα-mediated transcription and suppress proliferation. Under dual hormone stimulation, AR is redirected to ERE-enriched regions, where it remodels ERα chromatin engagement and represses oncogenic targets such as *MYB*, *MYC*, and *CCND1*. Simultaneously, agonist-bound AR reprograms ERα activity by assembling an AR/ERα/p300/SRC-3 coactivator complex at AREs, thereby promoting transcription of AR target genes with putative tumor-suppressive functions, including SEC14L2 and ZBTB16^347^.

AR signaling functions as a molecular switch in ERα+ breast cancer, transitioning from a tumor-suppressive to an oncogenic driver in a time-dependent manner. Short‑term AR activation exerts a tumor‑suppressive role by reprogramming the AR/ERα/FOXA1 cistrome, suppressing ER‑driven oncogenic programs, and enhancing luminal differentiation while reducing basal‑like features. In contrast, sustained AR activation rewires the cistrome with loss of canonical ARE/ERE binding and gain of PROP1 motifs, together with activation of JAK/STAT signaling, which drives EMT, lineage plasticity, and a basal‑like invasive phenotype^365^.

**Fig. 5:**
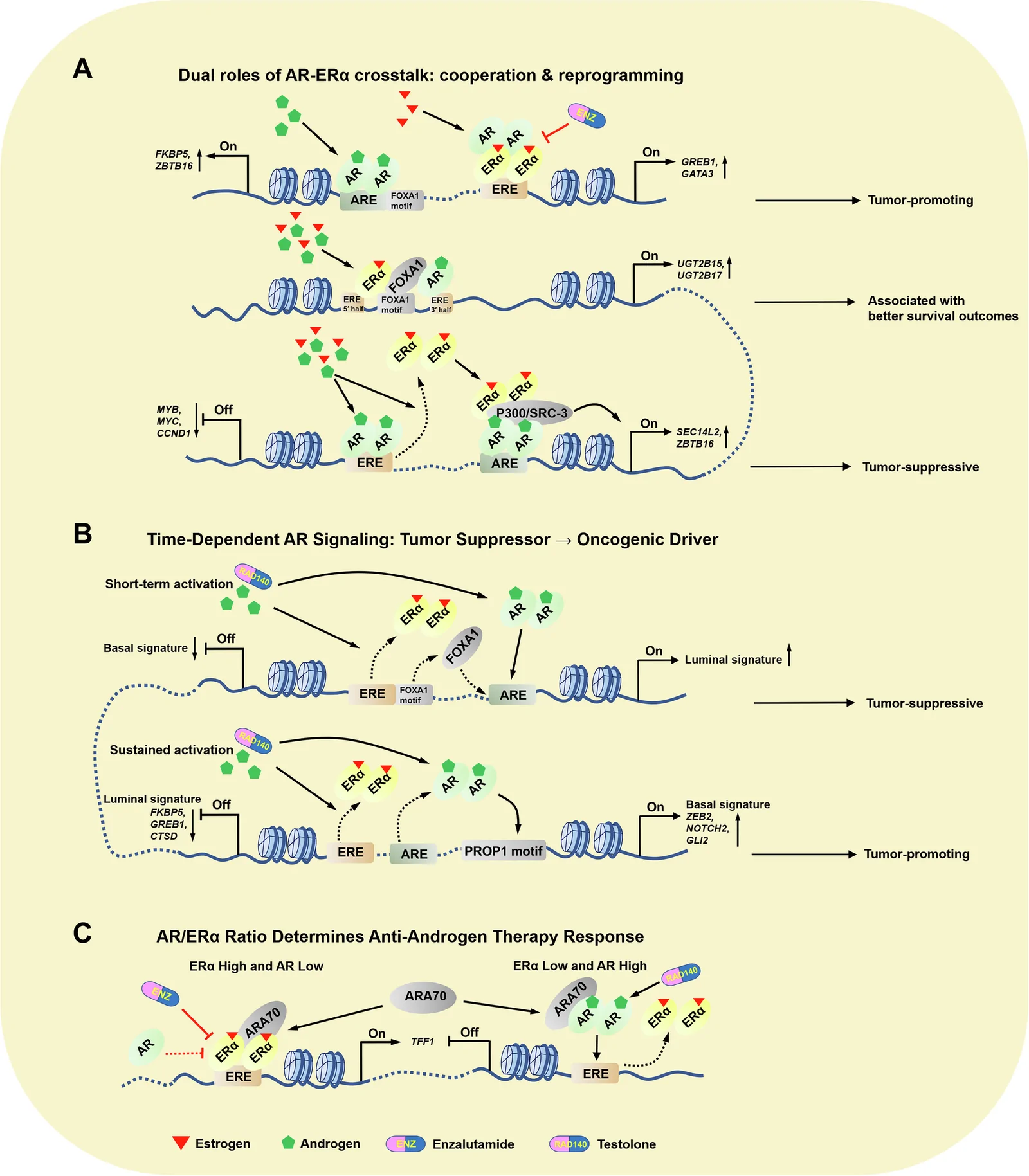
Switch of AR-ERα crosstalk and AR ligand functions in breast cancer. **A** DHT activates AR binding to a distinct FOXA1‑enriched cistrome, promoting transcription of canonical AR target genes such as FKBP5. In contrast, under estradiol stimulation, AR is redirected to ERE‑enriched loci, enhancing ERα binding and proliferative signaling; both interactions are disrupted by enzalutamide, which synergizes with tamoxifen and fulvestrant to suppress tumor growth; AR, ERα, and FOXA1 co‑occupy composite elements to regulate steroid‑metabolizing enzymes (UGT2B15/17), correlating with favorable prognosis; Conversely, AR activation can antagonize ERα transcription, repress oncogenic targets (MYB, MYC, CCND1), and induce tumor‑suppressive programs (e.g., SEC14L2, ZBTB16), in part by reprogramming ERα activity through assembly of an AR/ERα/p300/SRC‑3 coactivator complex. **B** AR acts as a time‑dependent molecular switch: short‑term activation suppresses ERα programs and promotes luminal features, whereas sustained activation reprograms the cistrome to drive basal‑like invasive phenotypes. **C** At low AR levels, ARA70 preferentially enhances ERα transcriptional activity, whereas at high AR levels, ARA70 shifts to reinforce AR‑mediated repression of ERα signaling; The selective AR agonist RAD140 (Testolone) is more effective in AR‑high models by consolidating AR’s antagonistic role, while the AR antagonist enzalutamide shows greater efficacy in AR‑low models, where its growth inhibition is mediated largely through interference with ERα signaling. ERE estrogen response element, ARE androgen response element, FOXA1 motif FOXA1-enriched DNA binding sequences, PROP1 motif PROP1-enriched DNA binding sequences, ENZ Enzalutamide. RAD140: Testolone.

AR exhibits context-dependent functions in ERα+ breast cancer, shaped by the AR/ERα ratio and coactivator availability. At low AR levels, the shared coactivator ARA70 (NCOA4) enhances ERα transcriptional activity and AR is insufficient to suppress ERα signaling. As AR expression increases and becomes dominant relative to ERα, ARA70 shifts to reinforce AR-mediated repression, enabling AR to interfere with ERα binding to ERE, suppress ERα target gene expression, and consolidate its antagonistic transcriptional role^366^. Both the selective AR agonist RAD140 and the AR antagonist enzalutamide exhibit therapeutic activity in ERα+ disease, with agonist (RAD140) preferentially suppressing growth in AR-high models and antagonist enzalutamide more effective in AR-low models; notably, in AR-low contexts, enzalutamide’s growth inhibition is likely mediated through ERα signaling rather than AR (Fig. 5)^367^.

Together, these findings highlight AR as a molecular switch and context-dependent modulator of ERα signaling in breast cancer. Depending on ligand availability, temporal dynamics, or the AR/ERα ratio, AR can either cooperate with ERα to promote proliferation, redirect ERα binding to repress oncogenic programs, or establish tumor-suppressive transcriptional outputs. This mechanistic plasticity not only explains the dualistic roles of AR in ERα + BCa but also provides a rationale for therapeutic strategies that exploit AR agonists or antagonists in distinct biological contexts.

### The effect of AR in ERα-negative BCa

AR is expressed in both ERα-/HER2+ breast cancer and the luminal androgen receptor (LAR) subtype of TNBC. In ERα-/HER2+ disease, AR frequently co-expresses with HER2, and the AR+/HER2+ phenotype represents an independent adverse prognostic factor associated with shortened disease-free survival^368^. Although LAR-TNBC, characterized by high AR expression, displays a lower proliferation rate and relatively favorable prognosis compared to other TNBC subtypes^369^, elevated AR expression across TNBC broadly remains negatively associated with progression-free and overall survival^370^. These studies suggest that AR represents an attractive therapeutic target. A number of clinical trials assessing AR + ERα- patients receiving second-generation AR antagonists have yielded encouraging outcomes. Moreover, the combination of AR inhibitors with other anti-cancer agents has the potential to enhance the therapeutic efficacy against AR + ERα- BCa, including chemotherapy agents, PARP inhibitors, HDAC inhibitors and CDK4/6 inhibitors^5,11,371–375^.

#### The carcinogenic role of AR

In ERα-/HER2+ breast cancer, AR is functionally active and collaborates with FOXA1 to establish a unique hormone-regulated cistrome that transcriptionally upregulates WNT7B and HER3, thereby activating oncogenic Wnt/β-catenin and HER2/HER3 signaling to drive androgen-dependent tumor growth; notably, the AR antagonist bicalutamide effectively blocks these pathways and inhibits tumor progression in vivo, positioning AR as an actionable therapeutic target in this subtype^348^. Similarly, AR and ERK constitute a positive feedback loop that drives ERα-/HER2+ molecular apocrine BCa cells proliferation. Accordingly, combined blockade with the AR antagonist flutamide and the MEK inhibitor exhibits marked synergistic anti-tumor activity both in vitro and in vivo, and can overcome resistance to the anti-HER2 monoclonal antibody trastuzumab^376^. Despite these identified oncogenic mechanisms, the independent functional and mechanistic landscape of AR specifically within the ERα-/HER2+ subtype remains comparatively underexplored, as AR-related research in ERα-negative breast cancer has focused predominantly on TNBC.

In the TNBC setting, the presence of AR was linked to poorer local recurrence-free survival and a heightened risk of locoregional recurrence following radiation treatment. Functional AR plays a crucial role in the efficient repair of double-stranded DNA breaks caused by ionizing radiation, while the suppression of AR activity notably hinders this repair process, partially via a mechanism involving the DNA protein kinase catalytic subunit (DNAPKcs)^377^. AR-mediated KLF4 transcription enhances BCa stem-like properties that are linked to chemotherapy resistance and metastatic recurrence^378^. Moreover, in TNBC cells (MDA-MB-231) and non-cancerous cells (MCF10A), the synthetic androgen methyltrienolone induces p21 transcription, which in turn activates MKP-1 phosphatase, which prevents MAPK dephosphorylation, leading to enhanced MAPK signaling and proliferation. In contrast, combined activation of EGFR and AR leads to sustained MAPK signaling, inducing senescence-like growth arrest. Furthermore, in cases where p21 is mutated or absent, AR loses its regulatory capacity over cell proliferation in ERα-PR- breast epithelial cells^379^.

#### The tumor-suppressive role of AR

However, several studies have demonstrated that the DHT-AR signaling pathway also inhibits the proliferation of AR-positive, ERα-negative BCa cells. Unlike in prostate cancer, DHT-activated AR upregulates the transcription of the tumor suppressor PTEN, leading to growth arrest in MDA-MB-453 cells^380^. Another study shows that AR-mediated KLLN upregulation enhances cell apoptosis through p53 and p73 induction^381^. A recent study has elucidated the dual role of AR in TNBC cells ferroptosis. On one hand, AR directly suppresses the transcription of DECR1, resulting in the accumulation of polyunsaturated fatty acids (PUFA) and thereby promoting ferroptosis. Conversely, AR enhances the transcription of GPX4, which induces the production of phosphoilpid peroxides and consequently inhibits ferroptosis^382^. These findings may provide partial insights into the paradoxical roles of AR in triple-negative BCa.

### Epigenetic co-regulation of AR-induced transcription in BCa

It has been reported that numerous epigenetic regulators participate in AR-mediated gene transcription in prostate cancer^383,384^, with some also acting as co-regulators of AR in BCa. For instance, KDM3A specifically demethylates histone H3K9me1/2 facilitating transcription activation mediated by AR^385^. DHT-activated AR drives EMT through coordinated epigenetic remodeling at E-cadherin and vimentin promoters. AR recruits LSD1 to remove H3K4me2 and facilitates SUV39H1‑mediated H3K9me2 deposition, thereby repressing E‑cadherin. Conversely, AR-LSD1 complexes reduce H3K9me2 and enhance MLL1-dependent H3K4me2 at the vimentin promoter, activating its transcription. These findings demonstrate that DHT promotes breast cancer invasion and metastasis through an LSD1‑dependent EMT program, independent of ER signaling^346^. Inhibition of CBP/p300 bromodomains suppresses the expression of super‑enhancer-associated genes that are enriched for AR binding in ERα‑negative, AR-positive breast cancer cells. These inhibitors reduce AR signaling pathway activity by decreasing H3K27ac levels at AR-binding sites^386^. The SIN3-HDAC1-Ebp1 complex effectively suppresses the expression of AR target genes, including PSA and E2F1A^387^. Histone deacetylase (HDAC) inhibitors have been employed in BCa management, and their combination with AR antagonists demonstrates greater therapeutic efficacy than monotherapy in LAR TNBC^375,388^.

Pioneer factors such as FOXA1 and GATA3 are involved in AR-mediated gene transcription. Reports indicate that silencing of FOXA1 diminishes AR binding to the promoter of target genes associated with cell proliferation^389^. In the absence of androgen stimulation, TCF7L2 and FOXA1 recruit co-repressors that inhibit the basal expression of the AR downstream target gene. GATA3 interacts with AR to transcriptionally regulate the differentiation of luminal epithelial cells in BCa, independent of ER status^390^. Upon androgen stimulation, AR upregulates the transcription of WNT7B and MYC. On one hand, WNT7B activates the Wnt/β-catenin signaling pathway, and β-catenin serves as a co-activator to enhance AR-mediated transcription of HER3, thereby activating the downstream kinase AKT signaling pathway. On the other hand, activated AKT facilitates the recruitment of MAX-MYC to the promoters of AR target genes, thereby enhancing AR signaling in a positive feedback loop^348,391^. Prostate-derived Ets factor (PDEF) is transcribed by AR and collaborates with AR signaling pathways to mediate the proliferation of ERα+, AR-positive BCa cells^392^.

### Post-translational modification of AR in BCa

PTM of AR are essential for both ligand-dependent and independent activation of the AR signaling pathway. Accumulating evidence suggests that regulatory PTM factors involved in AR-mediated downstream gene transcription play significant roles in BCa progression. The AR protein is influenced by various PTM, including phosphorylation, ubiquitination and SUMOylation^11^.

#### Phosphorylation

Similar to other nuclear receptors, the AR protein undergoes rapid phosphorylation through various kinase pathways, with more than a dozen serine, threonine, or tyrosine residues available for modification. Phosphorylation at distinct sites on the AR can modulate its transcriptional activity, stability, and cellular localization. The implications of phosphorylated AR in prostate cancer have been extensively studied and reviewed^393–395^. However, research on AR phosphorylation modifications in BCa remains limited. Nuclear and cytoplasmic phosphorylated AR S213/210 was identified in a study of 379 BCa tissue samples. Nuclear positive expression of phosphorylated AR S213/210 correlated with lower PR and Ki67 levels. Higher nuclear phosphorylated AR S213/210 expression was linked to prolonged RFS, while no significant association was found between survival and cytoplasmic phosphorylated AR S213/210 expression^396^. It has been reported that high levels of AR S213 and S650 phosphorylation have been found in either ERα-negative or metastatic BCa, which may be a potential marker of poor prognosis^397^. Further investigation is required to elucidate the direct phosphatases that catalyze these residues of AR in BCa. Another research has demonstrated that Kindlin-2, a focal adhesion protein, physically interacts with AR and Src, thereby facilitating Src-mediated phosphorylation of AR at Tyr-534, which promotes the proliferation and migration of TNBC cells^398^ Furthermore, AR can modulate the activity of kinases and phosphatases, thereby influencing downstream signaling pathways. Mechanistically, AR transcriptionally upregulates ErbB2, leading to an increase in phosphorylated ERK levels; in turn, phosphorylated ERK enhances CREB1 expression to mediate AR transcription, thereby establishing a positive feedback loop^399^.

#### Ubiquitination and SUMOylation

Similar to other ligand-activated nuclear receptors, AR is subject to stringent regulation by the ubiquitin-mediated system. K845 and K847 in the ligand-binding domain of AR have been identified as two ubiquitination sites^400^. Research on AR ubiquitination in prostate cancer has been well established, with E3 ubiquitin ligases such as RNF6, MDM2, and CHIP catalyzing the ubiquitination of AR, while certain ubiquitin-specific protease (USP) family proteins facilitate its deubiquitination^401–403^. Research on the ubiquitination of AR in BCa is limited; However, investigations into the regulation of AR stability by deubiquitinase USP14 indicate that AR ubiquitination plays a significant role in BCa progression. It has been reported that USP14 inhibited K48 ubiquitination and proteasomal degradation of the AR protein, thereby promoting cell proliferation in AR-responsive ERα-negative BCa cells by facilitating the transition from G0/G1 to S phase^404^. Enzalutamide combined with USP14 inhibition significantly downregulated AR proteins and suppressed AR-related signaling pathways, including Wnt/β-catenin and PI3K/AKT, in both ERα+ and ERα-BCa^405^.

It has been reported that SUMO-modified AR (SUMO-AR) persists in endocrine therapy resistant BCa cell lines. AR is SUMOylated by HSPB1/Hsp27, which protects it from ubiquitin-mediated proteasomal degradation. Persistent AR consistently mediates a series of gene transcription events within the nucleus, thereby facilitating the induction of EMT^406^.

### AR variants

Among the AR splice variants, AR‑V7 is the most clinically relevant form; it lacks the LBD and consists only of the NTD, the DBD, and a unique 16‑amino‑acid C‑terminal sequence encoded by cryptic exon 3b^5^. AR‑V7 localizes constitutively to the nucleus irrespective of androgen availability, resulting in ligand‑independent transcriptional activity that is unresponsive to LBD‑targeted therapies such as enzalutamide and abiraterone, an insensitivity well established in prostate cancer and increasingly recognized in breast cancer^407,408^.

Across studies, AR‑V7 is detectable in a substantial subset of breast cancers, with marked enrichment in ERα‑negative subtypes. AR‑V7 was identified in 18% of TNBC, 15.4% of HER2‑positive tumors, and 3.7% of ERα+/HER2 tumors, with two-thirds of all AR-V7-positive cases classified as TNBC. In addition, AR‑V7 was detected in 41% of circulating tumor cell samples from ERα‑positive metastatic breast cancer, with enrichment in cases showing bone‑predominant metastatic progression^361,409–411^. AR-V7-positive tumors frequently exhibit apocrine morphology, suggesting a potential histologic indicator for case selection when AR‑targeted therapy is being considered^410^.

Microarray profiling demonstrated that AR‑V7 in breast cancer regulates a transcriptional program enriched for immune‑related pathways, including interleukins, interferons, and chemokine signaling, as well as genes involved in cell motility. This contrasts with prostate cancer, where AR‑V7 predominantly regulates cell-cycle-associated genes, indicating that AR‑V7 activates a breast cancer specific transcriptional network rather than recapitulating the canonical prostate AR‑V7 program^409^. Functionally, AR‑V7 promotes a more proliferative phenotype in breast cancer cells and can persist independently of full‑length AR^412^.

AR‑V7 has been detected in bone metastases from TNBC that relapsed after one year of adjuvant enzalutamide, and it can also be induced in breast cancer cell lines following enzalutamide exposure. These findings suggest that AR‑V7 may represent a mechanism of acquired resistance to AR‑targeted therapy^407,413^. Notably, however, the biological context of AR‑V7 in breast cancer appears to differ from that in prostate cancer. Whereas AR‑V7 upregulation in prostate cancer typically reflects an acquired resistance mechanism emerging after prolonged androgen‑deprivation therapy, AR‑V7 can be detected in both primary and metastatic breast tumors independent of prior endocrine treatment. This pattern indicates that, in breast cancer, AR‑V7 may represent an inherent molecular feature rather than a treatment‑induced resistance product^409,410^.

In LAR TNBC, AR-V7 promotes a more aggressive proliferative and migratory phenotype and is associated with enrichment of interferon and IL6-JAK-STAT signaling. STAT1 functions as an AR coactivator within this axis, and the NTD‑binding selective androgen receptor degrader (SARD) UT-105 degrades both full-length AR and AR-V7, thereby suppressing AR‑dependent transcription and JAK-STAT pathway activity^344^. EPI‑7170, an inhibitor of the AR N‑terminal domain, suppresses the transcriptional activity of both AR‑FL and AR‑V7. In AR‑expressing breast cancer cells, it impedes AR‑dependent G1-S progression, prolongs S‑phase transit, reduces cyclin A2 and RAD51 levels, and induces DNA damage, collectively impairing proliferation. Its inhibitory effects in AR‑V7-positive breast cancer cells exceed those of LBD‑targeted agents such as enzalutamide^408^. These findings highlight AR-V7 as both a driver of therapeutic resistance and a functional target that can be effectively suppressed by NTD directed agents. Additional strategies such as AR degradation, splicing inhibition, and targeting of the DNA binding domain may also offer therapeutic opportunities in AR-V7 positive breast cancer^414^.

Despite growing interest, the clinical utility of AR‑V7 in breast cancer remains limited. Existing studies are constrained by small and heterogeneous patient cohorts, inconsistent detection methods, and incomplete correlation with therapeutic outcomes. Non‑invasive detection strategies have not been systematically evaluated, and the predictive value of AR‑V7 for AR‑targeted therapy remains unconfirmed. Larger, well‑designed prospective studies are required to define the diagnostic and therapeutic relevance of AR‑V7 in breast cancer^345,409,410^.

Collectively, AR signaling in breast cancer is characterized by profound context‑dependent plasticity, acting as either a tumor suppressor or oncogenic driver depending on molecular context. Analogous to PR‑ERα interplay, AR‑ERα crosstalk represents a competitive, ratio‑dependent interaction for shared transcriptional co‑regulators and chromatin accessibility, serving as a molecular switch that reversibly converts between cooperative proliferation and antagonistic growth restraint. This functional duality is dictated by the relative abundance of AR versus ERα, ligand availability, and temporal activation dynamics, providing a framework for understanding its paradoxical subtype‑specific behaviors. Beyond these receptor interactions, epigenetic coregulators, post‑translational modifications, and ligand‑independent variants such as AR‑V7 add further layers of regulatory complexity. However, significant gaps persist, including limited mechanistic insights into AR function within ERα‑/HER2+ disease and a lack of biomarkers predicting transitions between tumor‑suppressive and oncogenic states. Addressing these unresolved aspects will be essential to translating AR into an effective target for precision combinatorial therapy.

## Shared versus receptor-specific roles of co-regulators across sexual hormone receptors

Sexual hormone receptors (ER, PR, and AR) share highly conserved modular architectures that enable them to recruit an extensively overlapping repertoire of co-regulatory factors^3,18^. In the agonist-bound state, these receptors preferentially engage coactivators, including but not limited to p160/SRC family (SRC-1/2/3), histone acetyltransferases CBP/p300, arginine methyltransferases CARM1 and PRMT1, and ATP-dependent chromatin remodeling complexes such as SWI/SNF^21,169,322,415–417^, thereby activating transcription through epigenetic mechanisms. Conversely, in the unliganded or antagonist-bound state, receptors switch to recruiting corepressor complexes such as NCoR/SMRT and HDACs^126,127,180,418,419^, which maintain chromatin compaction and transcriptional silencing. This competitive equilibrium and cyclical assembly between coactivators and corepressors represents a universal principle of nuclear receptor signaling^211,212,417,420^.

Nevertheless, these broadly shared co-regulators do not function as interchangeable modules across receptors; their functional output is critically shaped by receptor conformation, docking interfaces, and cellular context^19^. For example, the p160/SRC family engages ERα and PR primarily through LxxLL motifs docking into the AF-2 hydrophobic cleft. MUC1 associates with ERα and facilitates recruitment of SRC-1/2, leading to increased transcriptional activity^170^. Phosphorylated SRC2 enhances its interaction with CBP/p300, thereby strengthening coactivation of ERα, PR. Similarly, estrogen-induced phosphorylation of SRC-3 promotes binding to p300 and supports ERα- and PR-dependent transcription. In contrast, full-length AR employs a fundamentally distinct coactivator assembly mechanism. Cryo-EM structures reveal that AR forms an asymmetric head-to-head and tail-to-tail dimer in which the two NTDs envelop the central LBDs, recruiting only a single SRC-3 molecule and binding p300 directly through the NTDs independent of the AF-2/LXXLL interface. Additional regulatory inputs, such as phosphorylation of SRC members or association with scaffold proteins, further tune coactivator recruitment for ERα and PR^170,421^. This NTD-centric recruitment, orchestrated by intra- and inter-molecular N/C interactions, underscores that identical coactivator can be deployed through divergent receptor architectures to elicit distinct transcriptional outputs^169,422^.

Although CBP/p300 serves as a shared coactivator for all three receptors, its functional output is receptor-specific. In ERα signaling, p300 enhances transcriptional activity through a dual mechanism involving direct acetylation of ERα and histone acetylation at target gene promoters, thereby cooperatively upregulating oncogenic gene expression^124,254^. Conversely, in ERα+ breast cancer, activated PR redirects ERα away from classical estrogen response elements to novel chromatin loci and co-recruits p300 to activate a tumor-suppressive gene program associated with good clinical outcome^300^. Similarly, AR can competitively sequester ERα co-regulators including p300 and SRC-3, thereby blocking ERα transcriptional activity and exerting a tumor suppressor function^347^. Likewise, other broadly shared chromatin regulators exemplified by pioneer factor FOXA1 and the SWI/SNF component BRG1 serve divergent roles across ERα, PR, and AR. Pioneer factor FOXA1 and BRG1 serve shared yet divergent roles across ERα, PR, and AR. FOXA1 preloads ERα and AR at active genes, but pre-marks silencing for PR. BRG1 enhances ERα accessibility within the BAF complex, yet PR recruits BRG1 independently of BAF to compact chromatin^328^.

Shared co-repressor and silencing complexes are differentially deployed across the three receptors. In the absence of agonist, all three recruit NCoR/SMRT-HDAC complexes to maintain chromatin compaction. As an example, antagonist-bound ERα selectively recruits NCoR and SMRT to mediate tamoxifen inhibition, and reduced NCoR levels correlate with acquired resistance^418^. By contrast, the unliganded PR assembles a distinct repressive complex represented by HP1γ/LSD1/CoREST/KDM5B complex that incorporates HDAC1/2^323^. For AR, coactivators and corepressors compete for a common surface within the N-terminal AF-1 domain. Agonists favor SRC-1 recruitment, whereas PR antagonist, mifepristone promote NCoR/SMRT binding via the N and C termini of AR to enforce repression^419,423^.

Enzymes regulating receptor ubiquitination exert distinct, receptor-specific effects. Representative E3 ligases such as E6-AP serves as both a coactivator and an E3 ubiquitin ligase, with these functions being separable; the C833S mutant retains coactivator capacity but loses ligase activity^210,424^. Across ERα, PR, and AR, hormone stimulation triggers E6-AP binding to enhance transcription while simultaneously driving receptor degradation, though tissue context shifts the emphasis. For ERα, E2-activated Src phosphorylates Y537 to recruit E6-AP, which then activates transcription while promoting ERα ubiquitination and degradation^425^. Regarding PR-B, E6-AP displays isoform specificity by degrading PR-B but sparing PR-A, thereby controlling mammary ductal branching^210^. For AR, E6-AP binds the N terminus to support transcription and mediate degradation. Its deletion elevates AR protein but lowers activity, causing p53 accumulation and prostate atrophy^426^. Another example, MDM2, illustrates further context-dependent divergence. In breast cancer cells, MDM2 both coactivates ERα transcription^208^ and mediates its p53-dependent ubiquitination and degradation^207^. Its relationship with PR remains unclear. For AR, MDM2 governs only degradation, with mechanisms varying by cellular state. In conventional prostate cancer cells, Akt-dependent phosphorylation activates MDM2 to promote AR ubiquitination and degradation, suppressing transcriptional activity^427^. In prostate cancer stem-like cells, MDM2 maintains an AR-negative phenotype through constitutive ubiquitination and proteasomal degradation of full-length AR, preserving stem cell self-renewal and proliferation^428^.

As a dual-specificity demethylase for H3K4 and H3K9, LSD1 exerts receptor-context-dependent functions in transcriptional regulation. In the ERα pathway, LSD1 promotes activation by switching substrate preference toward H3K9me2 upon PELP1 recruitment and coordinating removal of repressive H3K9me2/H3K27me3 alongside H3K4me3 establishment within the KMT2B-KDM6A complex^156,157^. Conversely, it can also repress transcription in a demethylase-independent manner by elevating H3K9me2^158^ or reverse SMYD2-mediated ERα inhibition to restore chromatin recruitment^263^. For PR, LSD1 participates in ligand-independent silencing via an SRA-nucleated repressive complex containing HP1γ, HDAC1/2, CoREST, and KDM5B^323^, whereas ligand-activated PR redirects LSD1 toward SUV39H2-mediated H3K9me3 deposition to establish facultative heterochromatin^323,328^. In the AR pathway, DHT-activated AR recruits LSD1 to bidirectionally remodel EMT loci, repressing E-cadherin through H3K4me2 demethylation and H3K9me2 deposition while activating vimentin via H3K9me2 removal and H3K4me2 enhancement, a process independent of ERα signaling^346^. Across all three receptors, LSD1 displays cofactor-dependent plasticity, regulates the H3K4/H3K9 methylation balance, and is recruited to receptor-bound regulatory elements; however, ERα primarily utilizes LSD1 for activation with context-specific repression, PR predominantly for repression, and AR for bidirectional control within the same cellular process, with substrate switching evident in ERα and AR but not reported for PR.

Our previous study identified MDC1 as a coactivator for sexual hormone receptor in both ERα-positive breast cancer and AR-positive prostate cancer, operating through similar mechanisms to exert tumor-suppressive effects^179,429^. In contrast, we found that BAP18 functions as a shared epigenetic coactivator for ERα and AR across both malignancies, enhancing receptor-mediated transcription and supporting malignant cell proliferation. Nevertheless, the functional contexts differ markedly: in ERα-positive breast cancer, BAP18 primarily drives enhancer transactivation and chromatin accessibility to facilitate estrogen-dependent gene expression, whereas in prostate cancer, it amplifies androgen signaling and sustains tumor growth even under androgen-depleted conditions. These distinct receptor-specific and cancer-specific roles demonstrate that although BAP18 universally amplifies hormone receptor signaling, it produces divergent oncogenic outputs and therapeutic vulnerabilities in different tumor contexts^176,430^.

In essence, the shared utilization of broad-acting co-regulators by ERα, PR, and AR provides a unified mechanistic basis for ligand-responsive transcription, whereas receptor-specific docking interfaces, differential complex composition, and contextual switching generate distinct functional outputs. This convergence of a common co-regulator repertoire with receptor-specific assembly mechanisms and context-dependent functional modulation ensures precise and adaptable control of gene expression, enabling identical hormonal cues to be translated into distinct receptor-specific and cell-type-specific transcriptional programs essential for both normal physiology and disease progression.

## The therapeutic potential of targeting hormone receptor co-regulators in BCa

Previous studies have demonstrated that endocrine resistance is closely associated with various epigenetic alterations, including changes in DNA methylation patterns, chromatin accessibility, histone modifications, and the binding of diverse transcription factors. Several small-molecule epigenetic inhibitors have been investigated for their potential to restore sensitivity of BCa cells and tumors to endocrine therapy^120^. The following section summarizes representative compounds, categorized by their molecular targets, that have been demonstrated or are currently being evaluated for inhibiting the progression of ERα + BCa or overcoming endocrine resistance (Table 2).

**Table 2:** Inhibitors targeting ERα co‑regulators in breast cancer therapy

Drug name	Target protein	Target classification	Drug type	Development stage in BCa	Combination use	References
Azacitidine	DNMT1/3A/3B	DNA methyltransferase	Small molecule inhibitor	Phase II	Yes (with Entinostat)	^433^
Decitabine	DNMT1/3A/3B	DNA methyltransferase	Small molecule inhibitor	Phase II	Single (endocrine-resistant BCa)	^431,432^
					Yes (with pembrolizumab in TNBC)	
Vorinostat	HDAC1/2/3/8	HDAC Class I/II	Small molecule inhibitor	Phase II	Yes (with Tamoxifen)	^434^
Trichostatin A	HDAC1/2/3/8	HDAC Class I/II	Small molecule inhibitor	Preclinical	Yes (with Tamoxifen)	^435^
Belinostat	HDAC1/2/3/8/10	HDAC Class I/II/IV	Small molecule inhibitor	Preclinical	Yes (with Everolimus + Paclitaxel + Pictilisib)	^436^
Panobinostat	HDAC1/2/3/8/10	HDAC Class I/II/IV	Small molecule inhibitor	Phase I	Yes (with Letrozole)	^439^
Tucidinostat	HDAC1/2/3/8	HDAC Class I/IIb	Small molecule inhibitor	Phase III	Yes (with Exemestane)	^131^
Entinostat	HDAC1/2/3	HDAC Class I	Small molecule inhibitor	Phase III	Yes (with Exemestane)	^130,440,441^
Romidepsin	HDAC1/2	HDAC Class I	Small molecule inhibitor	Preclinical	Yes (with Cisplatin)	^442^
MHY2256	SIRT1	Sirtuin (HDAC Class III)	Small molecule inhibitor	Preclinical	Single	^437^
Selisistat	SIRT1	Sirtuin (HDAC Class III)	Small molecule inhibitor	Preclinical	Yes (with Paclitaxel)	^443^
C646	p300	HAT	Small molecule inhibitor	Preclinical	Single	^444^
Anacardic acid	p300/PCAF	HAT	Natural product inhibitor	Preclinical	Single	^448^
A-485	CBP/p300	HAT	Small molecule inhibitor	Preclinical	Single	^123,445^
CPI-1612	CBP/p300	HAT	Small molecule inhibitor	Preclinical	Single	^446^
CTx-648 (PF-9363)	KAT6A/B	HAT	Small molecule inhibitor	Preclinical	Single	^122^
PF-07248144	KAT6A/B	HAT	Small molecule inhibitor	Phase I	Single	^447^
Tazemetostat/ GSK343	EZH2	KMT	Small molecule inhibitor	Preclinical	Single	^141^
MS177/MS8815	EZH2	KMT	PROTAC degrader	Preclinical	Single	^451^
OICR-9429	WDR5	KMT subunit	Small molecule inhibitor	Preclinical	Yes (with Fulvestrant)	^452^
DZNep	SAHH & NSD2	Hydrolase (directly) KMT (indirectly)	Small molecule inhibitor	Preclinical	Single	^143^
UNC0642/BIX-01294a	G9a	KMT	Small molecule inhibitor	Preclinical	Single	^453,454^
Cyproheptadine	SET7/9	KMT	Small molecule inhibitor	Preclinical	Single	^455^
AZ506	SMYD2	KMT	Small molecule inhibitor	Preclinical	Yes (with Fulvestrant + Alpelisib)	^476^
iPRMT1	PRMT1	PRMT	Small molecule inhibitor	Preclinical	Single	^456,457^
QC6352	KDM4	KDM	Small molecule inhibitor	Preclinical	Single	^458^
KDM5-C70/C48/C49	KDM5	KDM	Small molecule inhibitor	Preclinical	Yes (with Fulvestrant)	^154^
Arformoterol	LSD1	KDM	Small molecule inhibitor	Preclinical	Single	^459^
Phenelzine	LSD1	KDM	Small molecule inhibitor	Phase I	Yes (with Nab-paclitaxel)	^460^
GSKJ4	KDM6A/B	KDM	Small molecule inhibitor	Preclinical	Single	^461^
Imatinib	c-Abl	Kinase	Small molecule inhibitor	Phase II	Yes (with Letrozole)	^465^
Samuraciclib	CDK7	Kinase	Small molecule inhibitor	Phase I	Single	^466^
THZ1	CDK7	Kinase	Small molecule inhibitor	Preclinical	Single	^60,61^
SY-1365	CDK7	Kinase	Small molecule inhibitor	Preclinical	Single	^467^
Capivasertib	Akt	Kinase	Small molecule inhibitor	FDA-approved	Yes (with Fulvestrant)	^468,469^
Selumetinib	MEK1/2	Kinase	Small molecule inhibitor	phase II	Yes (with Fulvestrant)	^471^
Trametinib	MEK1/2	Kinase	Small molecule inhibitor	Preclinical	Single	^470^
Bosutinib	Src/Abl	Kinase	Small molecule inhibitor	Phase II	Yes (with chemotherapy or AIs)	^472–474^
GNE-6776	USP7	DUB	Small molecule inhibitor	Preclinical	Single	^475^
DC-U4106	USP8	DUB	Small molecule inhibitor	Preclinical	Single	^217^
b-AP15/PtPT	USP14/UCHL5	DUB	Small molecule inhibitor	Preclinical	Single	^218^
Thiolutin	PSMD14	DUB	Small molecule inhibitor	Preclinical	Single	^182^
MLN4924	CRL4B	Ubiquitin ligase modulator	Small molecule inhibitor	Preclinical	Yes (with Tamoxifen)	^87^
TAK-981	SAE1/UBA2	SUMO E1	Small molecule inhibitor	Phase I	Single	^477^
Ginkgolic acid	SAE1/UBA2	SUMO E1	Natural product	Preclinical	Single	^478^
Spectomycin B1	UBC9	SUMO E2	Small molecule inhibitor	Preclinical	Single	^479^
BY13	SRC-3	Epigenetic regulator	PROTAC degrader	Preclinical	Single	^481^
PiET	BRD4/JMJD6	Epigenetic regulator	Peptide inhibitor	Preclinical	Single	^482^
9f (FL-411)	BRD4	Epigenetic regulator	Small molecule inhibitor	Preclinical	Single	^483^
dTRIM24	TRIM24	Epigenetic regulator	PROTAC degrader	Preclinical	Single	^449^
Onapristone	PR	Nuclear receptor	Antagonist	Phase II	Yes (with Fulvestrant)	^484^
Telapristone	PR	Nuclear receptor	SPRM	Phase II	Single	^485^
Ulipristal acetate	PR	Nuclear receptor	SPRM	Preclinical	Single	^486^
Enobosarm	AR	Nuclear receptor	SARM	Phase II	Single	^355^
Testolone	AR	Nuclear receptor	SARM	Phase I	Yes (with Palbociclib)	^487,488^
Bicalutamide	AR	Nuclear receptor	Antagonist	Phase II	Yes (with AIs)	^489,490^
Enzalutamide	AR	Nuclear receptor	Antagonist	Phase II	Yes (with Fulvestrant)	^491^
Abiraterone	CYP17A1	Steroidogenic enzyme	Inhibitor	Phase II	Yes (with Prednisone or Prednisone +AI)	^492,493^
Seviteronel	CYP17 + AR	Steroidogenic enzyme + Nuclear receptor	Inhibitor/antagonist	Phase I	Single	^494^

Letrozole/Exemestane Aromatase inhibitor, Pembrolizumab PD‑1 antibody, Everolimus mTOR inhibitor, Paclitaxel/Nab‑paclitaxel/Cisplatin Chemotherapeutic agent, Pictilisib/Alpelisib PI3K inhibitor, Palbociclib CDK4/6 inhibitor, Prednisone Glucocorticoid

*DNMT* DNA Methyltransferase, *HDAC* Histone Deacetylase, *HAT* Histone Acetyltransferase, *KMT* Lysine Methyltransferase, *PROTAC* Proteolysis Targeting Chimera, *PRMT* Protein Arginine Methyltransferase, *KDM* Lysine Demethylase, *AI* Aromatase Inhibitor, *USP* Ubiquitin-specific protease, *DUB* Deubiquitinating enzyme, *SUMO E1* Small Ubiquitin-like ModifiERα-activating enzyme, *SUMO E2* SUMO-conjugating enzyme, *SPRM* Selective Progesterone Receptor Modulator, *SARM* Selective Androgen Receptor Modulator, *CYP17A1* Cytochrome P450 17A1.

### DNMT inhibitors

Azacitidine and decitabine, as DNA methyltransferase inhibitors, are FDA‑approved for the treatment of hematological malignancies such as MDS and AML, and have shown promising therapeutic activity in solid tumors. Accumulating evidence suggests their potential in ERα-positive BCa, particularly in cases resistant to endocrine therapy. Decitabine (5‑Aza‑2′‑deoxycytidine) inhibits DNMT1, DNMT3A, and DNMT3B, leading to global DNA hypomethylation that reprograms enhancer-promoter interactions and reshapes the 3D epigenome, thereby modulating ERα transcriptional programs and suppressing endocrine‑resistant ERα+ breast cancer. Furthermore, clinical evidence from a phase II neoadjuvant window trial (NCT02957968) indicates that combining decitabine with immune checkpoint inhibition (pembrolizumab) may extend therapeutic benefit to patients with locally advanced HER2‑negative breast cancer^431,432^. A multicenter phase II study demonstrated that 5-azacitidine achieved one partial response in women with advanced hormone-resistant BCa^433^.

### HDAC and HAT inhibitors

Histone deacetylase inhibitors (HDACis), functioning as epigenetic modulators, demonstrate antitumor activity in BCa models when used as monotherapy. Their mechanisms of action include remodeling chromatin structure, disrupting DNA damage response pathways, and restoring hormone receptor expression. For example, in a phase II clinical trial, the HDAC inhibitor vorinostat (Class I/II) combined with tamoxifen demonstrated the potential to reverse endocrine resistance in patients with ERα+ metastatic BCa, achieving a clinical benefit rate of ~40%^434^. In addition, trichostatin A (targeting classes I and II) may enhance the responsiveness of ERα-negative, hormone therapy-resistant BCa cells to tamoxifen by upregulating ERβ activity^435^. Moreover, belinostat (targeting classes I, II, and IV) enhances histone acetylation and DNA damage responses to upregulate ERα expression, thereby sensitizing BCa cells to endocrine therapy and showing potential to overcome resistance and suppress tumor growth in combination regimens^436^. Complementing these findings, the SIRT inhibitor MHY2256 is capable of inducing autophagic cell death in ERα-positive BCa cells^437^.

Building on their single-agent activity, histone deacetylase inhibitors (HDACis), when combined with conventional endocrine therapies or chemotherapeutic agents, can further enhance cytotoxic effects and help reverse acquired resistance. Both preclinical and early clinical evidence indicate that such combination regimens not only delay tumor progression but also restore sensitivity to aromatase inhibitors (AIs) and potentiate the efficacy of chemotherapy. For instance, panobinostat, a broad‑spectrum HDAC inhibitor targeting classes I, II, and IV, acts as a potent inhibitor of aromatase expression. In a phase I study (NCT01105312), its combination with the aromatase inhibitor letrozole demonstrated synergistic effects in suppressing the proliferation of hormone‑responsive breast cancer cells^438,439^. Similarly, tucidinostat (targeting classes I and IIb) has been shown to enhance the efficacy of exemestane in patients with advanced ERα-positive BCa who have progressed following endocrine therapy (NCT02482753)^131^. Entinostat, a class I HDACi, has also exhibited promising results. When combined with letrozole or exemestane, it significantly slowed tumor growth compared to either AI alone^440^. Its therapeutic potential was further evaluated in a phase II clinical trial (NCT00676663) involving patients with advanced ERα-positive BCa^130^. However, subsequent phase III data (NCT02115282) revealed that the addition of entinostat to exemestane failed to overcome therapeutic resistance^441^. These findings suggest that further stratification of responsive patient populations or optimization of combination strategies may be warranted. Moreover, the combination of romidepsin (class I HDACi) with the chemotherapeutic agent cisplatin has been shown to induce cell death and reduce drug resistance in BCa cells, irrespective of ER status, indicating its potential as a promising therapeutic approach^442^. In addition, selisistat, a selective SIRT1 inhibitor, enhances the antitumor efficacy of paclitaxel, thereby inhibiting the proliferation of ERα-positive breast cancer cells^443^. These results support the rationale for combining SIRT1 inhibition with chemotherapy to synergistically amplify therapeutic outcomes.

Accumulating pre-clinical and early clinical evidence demonstrates that small molecule inhibitors targeting HATs exert inhibitory effects on BCa proliferation and overcome endocrine resistance. C646 is a selective and competitive histone acetyltransferase p300 inhibitor that markedly inhibits the proliferation of human breast ductal carcinoma T47D cells and human BCa MCF7 cells^444^. The CBP/p300 inhibitor A-485 suppresses H3K27 acetylation at ERα target enhancers, blocks estrogen-regulated transcription, and induces senescence, thereby exerting antiproliferative activity against both wild-type and tamoxifen-resistant MCF7 cells^123,445^. The CBP/p300-selective inhibitor CPI-1612 potently suppresses ERα-dependent transcription and effectively inhibits the growth of BCa cell line models^446^. CTx-648 (PF-9363), a highly potent and selective KAT6A/KAT6B inhibitor, demonstrates robust anti-tumor activity that correlates with H3K23Ac inhibition in KAT6A-overexpressing, ERα-positive BCa models, including those resistant to endocrine therapy^122^. In a Phase I clinical trial (NCT04606446), PF-07248144, a novel KAT6A/KAT6B inhibitor, exhibited a generally tolerable safety profile and sustained antitumor activity in patients with heavily pretreated ERα-positive metastatic breast cancer^447^. Anacardic acid, a naturally occurring histone acetyltransferase inhibitor, suppresses the HAT activity of both p300 and PCAF, blocks ERα-ERE binding, and consequently inhibits ERα-mediated transcriptional responses^448^. The TRIM24 PROTAC (proteolysis-targeting chimeras) degrader (dTRIM24) effectively inhibits ERα-driven transcriptional activity by decreasing histone H3K23ac and H3K27ac, thereby demonstrating therapeutic efficacy in endocrine-responsive BCa models^449^.

### KMT and KDM inhibitors

Methyltransferase and demethylase inhibitors are emerging as highly promising therapeutic tools in oncology, offering precise epigenetic modulation that can reverse aberrant gene expression driving tumorigenesis and progression^450^. KMT and KDM inhibitors, supported by extensive pre-clinical studies and emerging early-phase clinical trials, constitute a promising epigenetic strategy to overcome endocrine resistance and eradicate tumor stemness in BCa by reversing oncogenic methylation marks. Among these KMTs, EZH2 has emerged as a critical epigenetic regulator implicated in tamoxifen resistance. While EZH2 methyltransferase inhibitors (e.g., Tazemetostat and GSK343) suppress BCa growth by blocking enzymatic activity^141^, EZH2-targeting PROTACs such as MS177 and MS8815 offer a distinct therapeutic strategy by inducing complete proteasomal degradation of EZH2, thereby inhibiting cell proliferation, cell cycle progression, and acquired tamoxifen resistance^451^. OICR-9429, a small-molecule antagonist that disrupts the WDR5-KMTs interaction, reduces H3K4me3 levels at promoters of BCa stem cell (BCSC)-associated genes, suppresses BCSC-related gene expression and tumorsphere formation, and enhances the anticancer efficacy of fulvestrant^452^. Meanwhile, DZNep, an inhibitor of the H3K36me2 methyltransferase NSD2, has demonstrated potent tumor-suppressive effects and pro-apoptotic activity in tamoxifen-resistant BCa models, both in vitro and in xenograft systems^143^. Further targeting epigenetic regulators, the small-molecule G9a inhibitors UNC0642 and BIX-01294a reduce H3K9me2 levels at target promoters, inhibit BCa cell proliferation and migration in vitro, and suppress tumor growth in vivo^453,454^. Notably, cyproheptadine binds to the substrate-binding site of SET7/9, thereby inhibiting ERα methylation at Lys302. This leads to proteasomal degradation of ERα and suppression of proliferation in estrogen-dependent MCF7 cells. Its efficacy in blocking estrogen-induced transcriptional activation and cell growth is comparable to that of tamoxifen monotherapy^455^. Finally, the PRMT1 inhibitor iPRMT1 selectively decreases H4R3me2a levels in MCF7 cells, contributing to the suppression of BCa cell proliferation^456,457^.

KDM inhibitors have demonstrated promising antitumor effects in BCa models. QC6352, a selective KDM4 inhibitor, suppresses the proliferation of BCa stem-like cells, as well as their sphere-forming capacity and xenograft tumor formation^458^. KDM5-C70 and KDM5-C48/C49 inhibit KDM5 activity, enhancing sensitivity to anti-estrogens by modulating ERα signaling and reducing transcriptomic heterogeneity^154^. Arformoterol, an LSD1 inhibitor, promotes apoptosis and induces G2/M cell cycle arrest in MCF-7 cells^459^. A phase I clinical trial (NCT03505528) evaluated the combination of LSD1 inhibitor phenelzine with nab-paclitaxel in patients with advanced or metastatic BCa^460^. GSKJ4 targets KDM6A/6B, leading to increased H3K27me3 levels and reduced BCa cell stemness^461^. Notably, metformin also elevates H3K27me3 by directly inhibiting KDM6A/B. Similarly, ibuprofen and aspirin suppress BCa cell stemness via KDM6A/6B inhibition, suggesting a shared epigenetic mechanism underlying their antitumor activity^462–464^.

### Kinase inhibitors

Kinase inhibitors continue to play a pivotal role in targeting endocrine-resistant BCa. The c-Abl inhibitor imatinib effectively suppresses BCa cell proliferation and invasiveness by inhibiting ERα phosphorylation and reducing ERα protein stability, and it has already advanced to phase II clinical trials^225,465^. A Phase I clinical study (NCT03363893) demonstrated that samuraciclib, a CDK7 inhibitor, exhibits antitumor activity in patients with advanced BCa^466^. Similarly, THZ1, another CDK7 inhibitor, inhibits the growth of MCF7 cells expressing either wild-type or mutant ER^60,61^. SY-1365, a highly selective covalent CDK7 inhibitor, exerts potent cytotoxic rather than merely cytostatic effects in ERα+ breast cancer through a dual-track mechanism: blocking cell cycle progression via CAK inhibition and suppressing transcriptional activity, particularly MYC and ER signaling. Notably, SY-1365 retains efficacy in models resistant to CDK4/6 inhibitors or harboring ESR1 mutations^467^. In a Phase III trial (NCT04305496), the Akt inhibitor capivasertib, when combined with fulvestrant, significantly prolonged progression-free survival compared to fulvestrant alone in patients with hormone receptor-positive advanced BCa, particularly those with prior aromatase inhibitor failure^468^. Capivasertib received FDA approval in 2023 for use in combination with fulvestrant to treat hormone receptor (HR)-positive, HER2-negative, locally advanced or metastatic BCa^469^. MEK1 inhibitors selumetinib (Phase II: NCT01160718.) and trametinib have also shown promise in overcoming antiestrogen resistance in ERα-positive luminal BCa^470,471^. Bosutinib, a Src/Abl tyrosine kinase inhibitor, has been evaluated in phase II trials (NCT00793546, NCT00880009) for BCa in patients pretreated with chemotherapy or in combination with aromatase inhibitors such as letrozole or exemestane for hormone receptor‑positive/HER2‑negative disease, highlighting its exploratory therapeutic potential^472–474^.

### DUB inhibitors

DUB inhibitors and related epigenetic modulators have emerged as promising therapeutic strategies in ERα-positive BCa. The USP7 inhibitor GNE-6776 induces apoptosis in ERα + BCa cells^475^. DC-U4106 targets USP8, promoting ERα degradation and significantly suppressing tumor growth with minimal toxicity^217^. Inhibitors of USP14 and UCHL5, including b-AP15 and platinum pyrithione (PtPT), also demonstrate growth-inhibitory effects in ERα + BCa models^218^. Thiolutin, a PSMD14 inhibitor, effectively restrains BCa progression^182^. The SMYD2 inhibitor AZ506 exhibits potent antitumor activity in ERα + BCa organoid models, both as monotherapy and in combination with fulvestrant and the PI3K inhibitor alpelisib, supporting its utility in multi-agent regimens^476^. Additionally, our previous findings revealed that imatinib, through docking to MYSM1, suppresses BCa cell proliferation by modulating the MYSM1-ERα signaling axis^168^. The CRL4B inhibitor MLN4924 enhances tamoxifen efficacy in resistant BCa cells by disrupting the CUL4B/miR-32-5p/ERα36 signaling pathway^87^.

### SUMOylation inhibitors

SUMOylation inhibitors have emerged as promising therapeutic agents in BCa by targeting distinct components of the SUMO cascade. In a phase I study, TAK‑981, a SUMO E1 inhibitor, demonstrated preliminary antitumor activity in a patient with relapsed HER2‑negative, hormone receptor‑positive breast cancer, highlighting its potential for subtype‑specific intervention^477^. Similarly, ginkgolic acid targets the E1-activating complex SAE1/UBA2, impairing cell cycle progression and inducing apoptosis in NOTCH1-driven breast epithelial cells^478^. Spectomycin B1 selectively binds to the E2-conjugating enzyme UBC9, effectively suppressing estrogen-dependent proliferation in hormone-sensitive BCa cells^479^.

### BRD4 and SRC-3 inhibitors

Other factor inhibitors have also shown potential in overcoming endocrine resistance and promoting BCa cell death^480^. SRC-3-targeting PROTACs, such as compound BY13, effectively degrade SRC-3 and suppress the proliferation of both wild-type and endocrine-resistant BCa cells^481^. In addition, PiET, a peptide inhibitor designed to target the extraterminal domain and disrupt the BRD4/JMJD6 interaction, has been shown to overcome tamoxifen resistance in ERα-positive BCa cells^482^. Complementing this, the small-molecule BRD4 inhibitor 9f (FL-411) induces ATG5-dependent autophagy-associated cell death, further supporting BRD4 as a therapeutic target in BCa^483^.

As noted earlier, the co‑regulator networks of PR or AR in breast cancer are far less comprehensively characterized than those of ERα, and effective pharmacologic strategies directly targeting PR‑ or AR‑specific co‑regulators remain limited. Moreover, although PR and AR signaling frequently exhibits crosstalk with the ERα pathway, therapeutic evidence in ERα‑positive breast cancer still largely derives from direct PR/AR modulators rather than co‑regulator‑targeting approaches, making these modulators a key focus in tumors with functional PR or AR expression.

### PR modulators

PR modulators have shown potential in luminal BCa, particularly in tumors with altered PR isoform expression. PR antagonist mifepristone significantly inhibits the proliferation of luminal BCa with a high PRA/PRB ratio, while activating immune and apoptotic pathways, supporting its use as a novel component in combined endocrine therapy^308^. A Phase II trial evaluating PR antagonist onapristone in combination with fulvestrant for ERα+/HER2− metastatic BCa reported good tolerability but limited antitumor efficacy^484^. As previously noted, pure antiprogestins may, under certain conditions, contribute to cancer progression. To address this limitation, selective progesterone receptor modulators (SPRMs) have been developed to achieve context-dependent, tissue-specific modulation of PR signaling. These compounds exhibit mixed agonist-antagonist activity and regulate PR-driven transcriptional programs. SPRMs have demonstrated preclinical efficacy in hormone-sensitive BCa^298^. In a randomized phase II presurgical window trial, telapristone acetate significantly reduced Ki67 levels in early-stage ERα+PR+ tumors, with marked antiproliferative effects and selective downregulation of HER2 amplicon‑associated genes in responsive cases^485^. In a preclinical model with conditional knockout of the BRCA1 C-terminal domain, ulipristal acetate (UPA) significantly reduced breast tumor incidence and extended tumor latency, highlighting its potential as a chemopreventive agent^486^.

### AR modulators

Interest in AR modulators has grown in breast cancer research, especially in subtypes that express functional AR. The SARM Enobosarm demonstrated antitumor activity in ERα+ advanced disease in a phase II trial (Study G200802), underscoring AR activation as a potential treatment strategy^355^. Similarly, the SARM RAD140 inhibited tumor growth in AR/ERα+ patient-derived xenograft models, with further enhancement observed in combination with the CDK4/6 inhibitor palbociclib. A phase I clinical trial additionally confirmed the safety of RAD140 and provided preliminary evidence of antitumor efficacy in AR+/ERα+/HER2− metastatic BCa^487,488^.

In the phase II trial (NCT02910050), bicalutamide combined with an aromatase inhibitor in ERα+AR+ advanced breast cancer achieved a high disease control rate and prolonged progression‑free survival, indicating that AR blockade can enhance endocrine therapy. In contrast, another phase II trial (NCT00468715), bicalutamide monotherapy in AR+ERα- metastatic breast cancer produced clinical benefit in only a subset of patients, with overall limited efficacy^489,490^. Enzalutamide has also demonstrated clinical benefit: in a Phase II trial (NCT02953860), its combination with fulvestrant achieved a 25% clinical benefit rate at 24 weeks in patients with metastatic ERα+HER2‑ breast cancer, while another phase II study (NCT01842321) showed activity in AR-positive TNBC, with benefit rates of 25% and 33% in the intention-to-treat and evaluable populations, respectively^33,491^.

The CYP17A1-lyase inhibitor abiraterone, combined with prednisone, achieved a 20% six-month clinical benefit rate and induced sustained remission in a subset of AR + TNBC patients (Phase II: NCT01842321). However, in ERα+ advanced BCa, abiraterone failed to improve progression-free survival, possibly due to elevated progesterone levels (NCT01381874)^492,493^. Seviteronel, a selective CYP17 lyase inhibitor and AR antagonist, showed modest activity in ERα-positive and TNBC patients, with four of seven achieving clinical benefit for four months (Phase I: NCT02580448)^494^.

## Conclusions and perspectives

Breast cancer remains a significant global health challenge due to its high incidence and considerable heterogeneity. Sexual hormone receptors, particularly the estrogen receptor (ER), progesterone receptor (PR), and androgen receptor (AR), play crucial roles in BCa pathogenesis and progression. This review provides a comprehensive overview of the structural and functional characteristics of these receptors, their intricate regulatory mechanisms, and the crosstalk between their signaling pathways.

The ER signaling pathway, primarily mediated by ERα, is a critical therapeutic target in BCa. However, endocrine resistance remains a significant challenge, often driven by genetic and epigenetic alterations in the *ESR1* gene. Recent studies have highlighted the importance of ERα variants, such as ERα46 and ERα36, in modulating therapeutic responses and contributing to resistance. Epigenetic modifications, including histone acetylation, methylation, and ubiquitination, as well as post-translational modifications of ERα, significantly influence its activity and stability. These findings underscore the complexity of ER signaling and suggest that targeting upstream regulators or downstream effectors of ERα may provide novel therapeutic strategies.

The PR signaling pathway exhibits dual roles in BCa, acting both as a tumor promoter and suppressor. PR isoforms, PRA and PRB, have distinct functions and regulatory mechanisms. PR can reprogram ERα-mediated gene transcription, influencing cell cycle progression and apoptosis. Epigenetic and post-translational modifications of PR, such as phosphorylation and SUMOylation, further complicate its role in BCa. Understanding the interplay between PR and ER signaling pathways is essential for developing more effective therapeutic approaches.

The AR signaling pathway also plays a complex role in BCa, with both tumor-suppressive and oncogenic functions. AR expression is associated with better outcomes in ERα-positive BCa and may predict response to endocrine therapies. However, AR overexpression can contribute to endocrine resistance and metastasis. Recent studies have shown that AR can inhibit ERα activity, suggesting its potential as a therapeutic target. Targeting AR signaling, either through androgen deprivation or AR antagonists, has shown promise in preclinical and clinical studies.

Future research should focus on elucidating the intricate crosstalk between these sexual hormone receptors and identifying novel therapeutic targets within their signaling pathways. Targeting co-regulators and epigenetic modifiers that influence receptor action may enhance the sensitivity of endocrine therapies in hormone receptor (HR) positive BCa. Combination therapies, such as pairing endocrine treatments with targeted agents against PR or AR, may provide synergistic effects to improve patient prognosis. Additionally, understanding the functions of sexual hormone receptor variants, post-translational modifications, and extranuclear signaling pathways will be crucial for developing more effective and personalized therapeutic strategies. Advances in molecular techniques, such as single-cell sequencing and CRISPR-based screening, will be invaluable in unraveling the complex regulatory networks driving BCa progression. Ultimately, a deeper understanding of these pathways will pave the way for innovative treatments and improved patient care.

## Data Availability

No datasets were generated or analyzed during the current study.
